# Supplementary material for: CellPhy: accurate and fast probabilistic inference of single-cell phylogenies from scDNA-seq data
Source: Genome Biol. 2022 Jan 26;23:37. doi: 10.1186/s13059-021-02583-w (PMC8790911; doi:10.1186/s13059-021-02583-w)
Supplement: Supplementary file 1 — Additional file 1. Supplementary Tables S1-S3, Figures S1-S22, and Notes S1-S3 [15, 21, 29, 51, 52, 66, 69, 73, 77, 91, 94–98]. [file 13059_2021_2583_MOESM1_ESM.pdf]

Additional file 1

CellPhy: accurate and fast probabilistic inference of single-cell phylogenies from scDNA-seq data

Alexey Kozlov, Joao Alves, Alexandros Stamatakis and David Posada

Contents

Table S1. Simulation scenarios.

Table S2. Genotype codes.

Table S3. CellPhy's compatibility with variant callers.

Fig. S1. Somatic diploid genotype phylogenetic model.

Fig. S2. Preliminary assessment of phylogenetic accuracy for all methods.

Fig. S3. Phylogenetic accuracy in Simulation 1 ("target-ISM") with 500 SNVs.

Fig. S4. Phylogenetic accuracy in Simulation 1 ("target-ISM") with 1,000 SNVs.

Fig. S5. Phylogenetic reconstruction accuracy in Simulation 3 ("WGS-sig") with signature S2.

Fig. S6. Phylogenetic reconstruction accuracy in Simulation 3 ("WGS-sig") with signature S6.

Fig. S7. Phylogenetic accuracy in Simulation 4 ("NGS-like") at 30x.

Fig. S8. Phylogenetic accuracy in Simulation 4 ("NGS-like") at 100x.

Fig. S9. Phylogenetic accuracy in Simulation 5 ("NGS-doublet").

Fig. S10. Estimation of the genotype error and ADO rate.

Fig. S11. Speed comparison with 100 bootstrap replicates using single or multi-threading.

Fig. S12. CRC24 genotype matrix for exonic variants.

Fig. S13. CRC24 variant allele frequency distribution in single-cell and bulk data.

Fig. S14. SiFIT, SCIPhI, infSCITE, ScisTree, and TNT trees for the CRC24 dataset.

Fig. S15. Non-synonymous mutations along the CellPhy L86 tree.

Fig. S16. SiFiT, SCIPhI, infSCITE, ScisTree, and TNT trees for the L86 dataset.

Fig. S18. Phylogenetic reconstruction from single-cell hematopoietic colonies.

Fig. S19. Genotype likelihood vectors for a simple unrooted phylogenetic tree.

Fig. S20. Phylogenetic likelihood calculations.

Fig. S21. Phylogenetic bootstrap calculation.

Fig. S22. Phylogenetic accuracy with approximate model GT10 in Simulation 6 ("NGS-large").

Supplementary Note 1. Genotype error model

Supplementary Note 2. Approximate model of evolution for unphased diploid genotypes with ten states

Supplementary Note 3. Standard phylogenetic likelihood calculations on DNA sequence alignments

Table S1. Simulation scenarios.

	**Sim 1 target-ISM**	**Sim 2 WGS-FSM**	**Sim 3 WGS-sig**	**Sim 4 NGS-like**	**Sim 5 NGS-doublet**	**Sim 6 NGS-large**
Number of ingroup cells	40	100	60	40	40	100, 500, 1000
Number of sites	5000	10000	10000	10000	10000	1000, 10000, 50000
Effective population size	10000	10000	10000	10000	10000	10000
Exponential growth rate	10^{-4}	10^{-4}	10^{-4}	10^{-4}	10^{-4}	10^{-4}
Root branch length	0.01	0.01	0.01	0.01	0.01	0.01
Outgroup branch length	0	0	0	0	0	0
Lineage rate variation (alpha)	1.0	1.0	1.0	1.0	1.0	1.0
Number of fixed SNVs	250, 500, 1000	n/a	n/a	n/a	n/a	n/a
Number of true SNVs	250, 500, 1000	1695 - 2560	1414 - 4215	1376 - 2123	1272 - 2094	181 - 347, 1841 - 3270, 7770 - 15705
Number of observed SNVs	206-4995, 415-4956, 807-4961	1531 - 10000	1224 - 10000	1147 - 10000, 1226 - 6841, 1227 - 2828	1271 - 9963	1000, 10000, 50000
Mutation rate	n/a	10^{-6}	10^{-6}	10^{-6}	10^{-6}	10^{-6}
Mutation model	ISM diploid	GTnR	ISM S2 and S6	GTnR	GTnR	GTnR
Mutation rate variation among sites (alpha)	n/a	1.0	n/a	1.0	1.0	1.0
Genotype error	0, 0.01, 0.05, 0.10	0, 0.01, 0.05, 0.10	0, 0.01, 0.10, 0.20	n/a	n/a	n/a

ADO	0, 0.10, 0.25, 0.50	0, 0.10, 0.25, 0.50	0, 0.05, 0.15, 0.50	0, 0.10, 0.25	0, 0.10	0.10
Sequencing depth	n/a	n/a	n/a	5, 30, 100	5	5
Coverage overdispersion	n/a	n/a	n/a	5	5	5
Sequencing error	n/a	n/a	n/a	0, 0.01, 0.05	0/0.01	0.01
Amplification error mean*	n/a	n/a	n/a	0, 0.05, 0.10	0/0.05	0.05
Amplification error variance	n/a	n/a	n/a	0.01	0.01	0.01
Allelic imbalance	n/a	n/a	n/a	0.50	0.50	0.50
Haploid coverage reduction	n/a	n/a	n/a	0.50	0.50	0.50
Doublet rate	0	0	0	0	0, 0.05, 0.10, 0.20	0
Number of replicates	100	100	100	100	100	20

Table S2. Genotype codes. Encoding of unphased genotypes as single letters for the FASTA/PHYLIP genotype files and translation to phased genotype likelihoods.

**Symbol**		**A**	**C**	**G**	**T**	**M**	**R**	**W**	**S**	**Y**	**K**
Genotype (unphased)		A/A	C/C	G/G	T/T	A/C	A/G	A/T	C/G	C/T	G/T
Genotypes (phased)		A|A	C|C	G|G	T|T	A|C<br>C|A	A|G<br>G|A	A|T<br>T|A	C|G<br>G|C	C|T<br>T|C	G|T<br>T|G
Phased genotype likelihood	A|A	**1**	0	0	0	0	0	0	0	0	0
	C|C	0	**1**	0	0	0	0	0	0	0	0
	G|G	0	0	**1**	0	0	0	0	0	0	0
	T|T	0	0	0	**1**	0	0	0	0	0	0
	A|C	0	0	0	0	**1**	0	0	0	0	0
	A|G	0	0	0	0	0	**1**	0	0	0	0
	A|T	0	0	0	0	0	0	**1**	0	0	0
	C|G	0	0	0	0	0	0	0	**1**	0	0
	C|T	0	0	0	0	0	0	0	0	**1**	0
	G|T	0	0	0	0	0	0	0	0	0	**1**
	C|A	0	0	0	0	**1**	0	0	0	0	0
	G|A	0	0	0	0	0	**1**	0	0	0	0
	T|A	0	0	0	0	0	0	**1**	0	0	0
	G|C	0	0	0	0	0	0	0	**1**	0	0
	T|C	0	0	0	0	0	0	0	0	**1**	0
	T|G	0	0	0	0	0	0	0	0	0	**1**

Table S3. CellPhy's compatibility with variant callers.

Algorithm	Single-cell specific	Multi-sample calling	Output format	CellPhy support		Reference
				ML mode	GL mode allowed	
SC-caller	Yes	No	(VCF)	Yes	Script	[66]
Monovar	Yes	Yes	VCF	Yes	Yes	[77]
Prosolo	Yes	No	BCF	Yes	Yes	[99]
Conbase	Yes	Yes	TSV	(Script)	No	[97]
sci-phi	Yes	Yes	(VCF)	Yes	No	[29]
scan-snv	Yes	No	RDA	(Script)	No	[98]
HaplotypeCaller	No	Yes	VCF	Yes	Yes	[91]

Remarks:

	Although not single-cell specific, HaplotypeCaller is still widely popular within the single-cell genomics community.
(VCF)	Non-standard VCF
Script	Conversion script provided with CellPhy
(Script)	Conversion possible with a custom script (not provided)

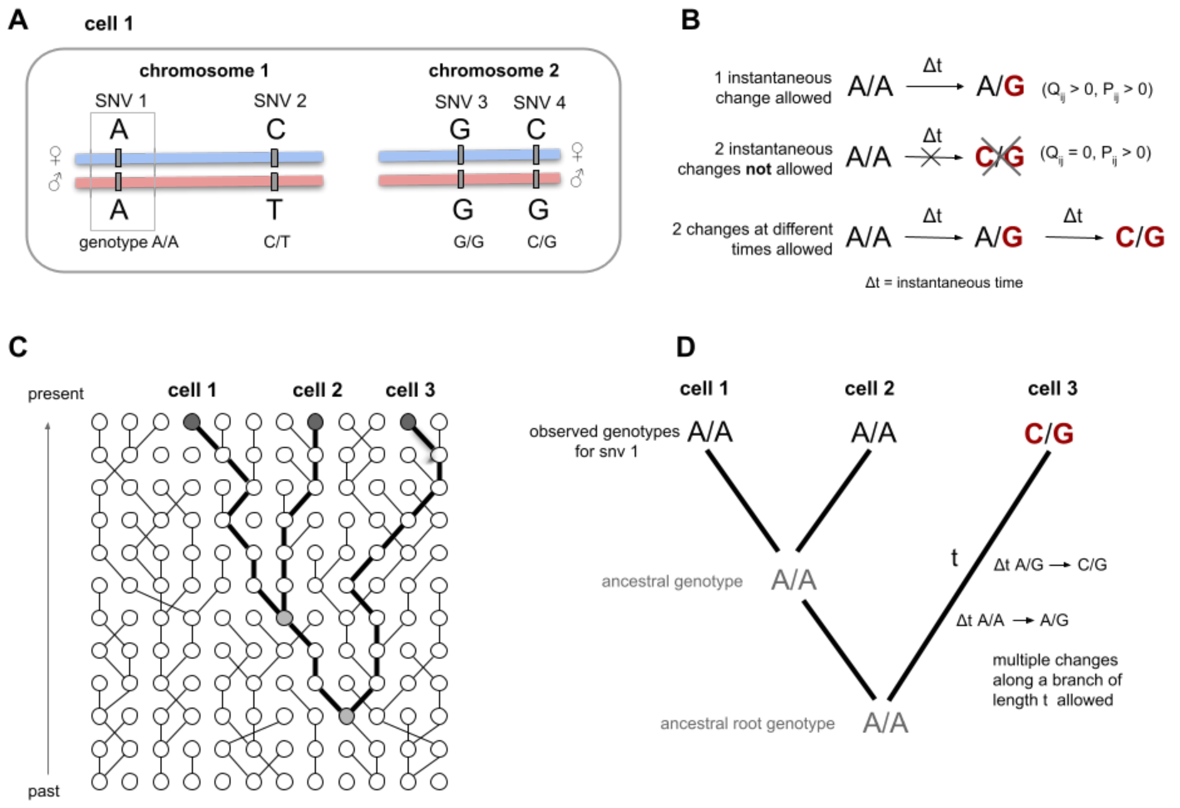

Fig. S1. Somatic diploid genotype phylogenetic model. A For each single nucleotide variable (SNV) site in the population, diploid genotypes are defined by the nucleotide present at the maternal (♀) and paternal (♂) homologous chromosomes. SNV sites are assumed to be independent, and their chromosomal position is ignored. **B** In a given infinitesimal amount of time (Δt), only one mutation is allowed in one of the two homologous chromosomes. These instantaneous rates form the Q matrix (Equation 2 in the main text) and contain 0 values when the corresponding genotypes differ at more than one nucleotide. **C** Three cells are sampled from a population of cells dividing asexually will have a specific genealogy (in bold). After cell division, cells will give rise to 0, 1, or 2 daughter cells (cells that die after division are not represented). **D** From the observed genotypes, we try to estimate the history of these three cells. Internal nodes are not observed, but the ancestral genotype at these nodes (in gray) can be estimated. More than one change can occur along any branch of length t (formed by infinite Δt). The transition probability matrix P(t) only considers the nucleotide at the beginning and at the end of a branch (Equation 3 in the main text), and all its entries are positive.

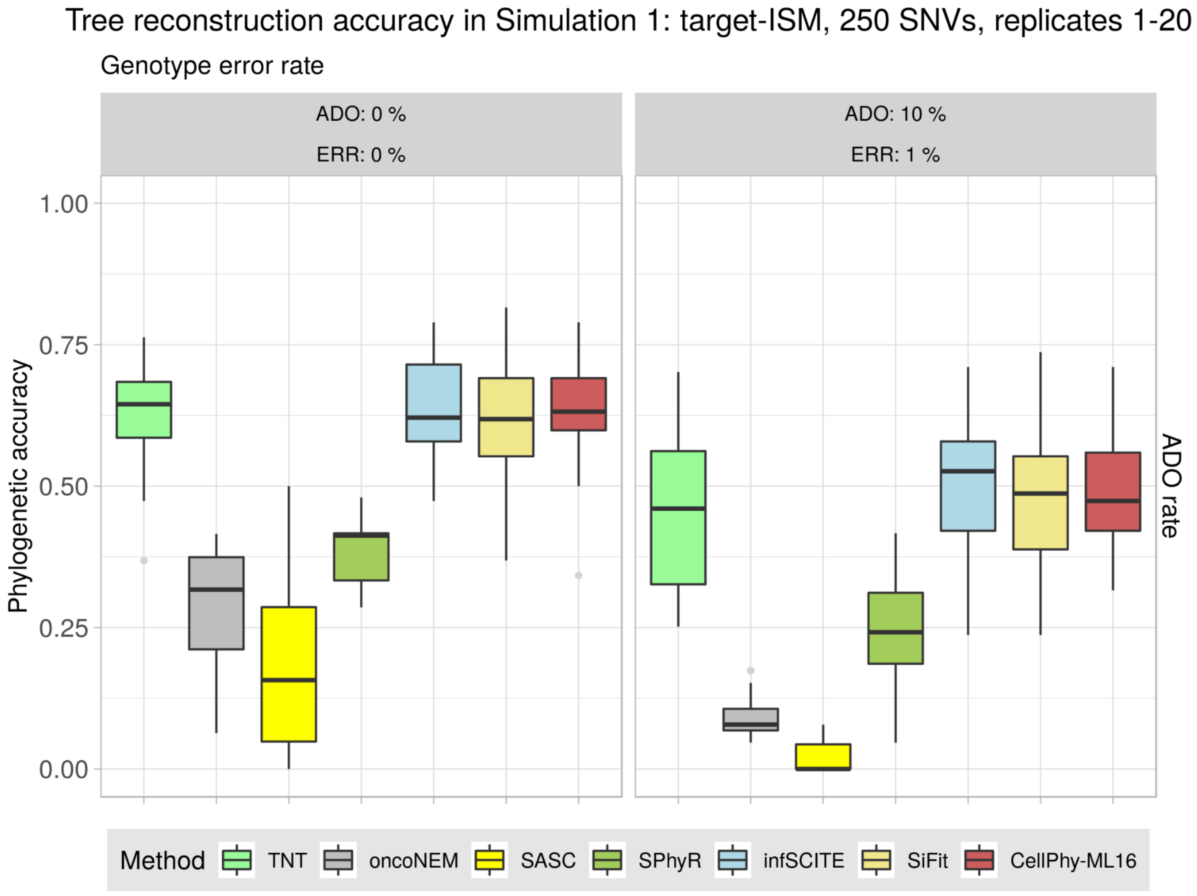

Fig. S2. Preliminary assessment of phylogenetic accuracy for all methods. Results for all methods were obtained for two scenarios of Simulation 1 (“target-ISM”). Datasets consisted of 40 tumor cells plus one healthy, with 250 SNVs. Accuracy was evaluated under two different levels of genotype error (ERR), allelic dropout (ADO), with the “missing” genotype recoding strategy. Phylogenetic accuracy is defined as 1 – nRF (see Methods). Results based on 20 replicates. Boxplots were generated using the ggplot2 R package (https://ggplot2.tidyverse.org) with default parameters. The lower and upper hinges correspond to the first and third quartiles. The upper whisker extends from the hinge to the largest value no further than 1.5 * IQR (IQR is the interquartile range or distance between the first and third quartiles). The lower whisker extends from the hinge to the smallest value at most 1.5 * IQR of the hinge. Data beyond the end of the whiskers are called "outlying" points and are plotted individually.

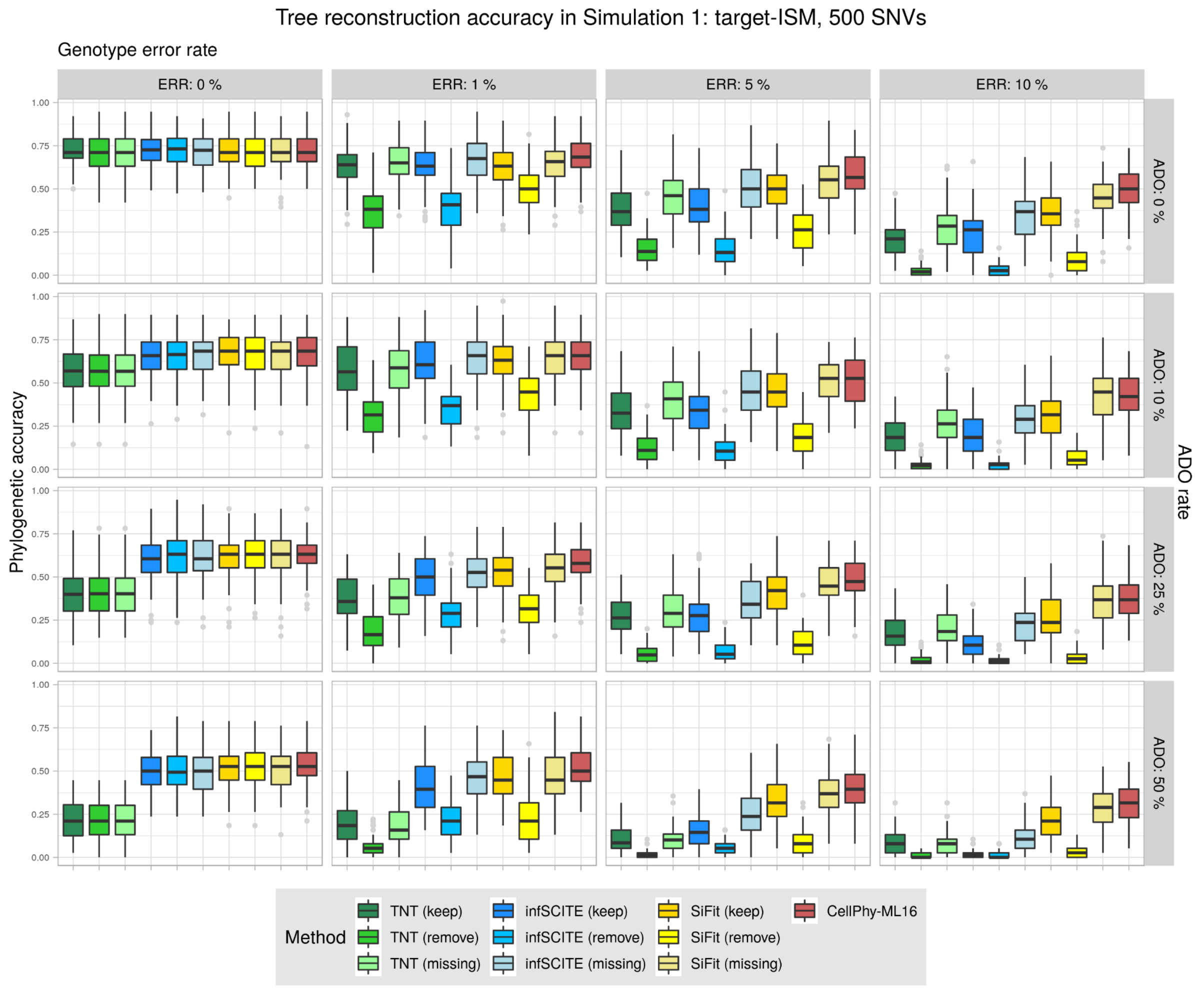

Fig. S3. Phylogenetic accuracy in Simulation 1 ("target-ISM") with 500 SNVs. Datasets consisted of 40 cells. Accuracy was evaluated under different levels of genotype error (ERR), allelic dropout (ADO), and genotype recoding strategies ("keep", "remove", "missing") as explained in the main text. Phylogenetic accuracy is defined as 1 – nRF (see Methods). See Fig. S2 for an explanation of the boxplots.

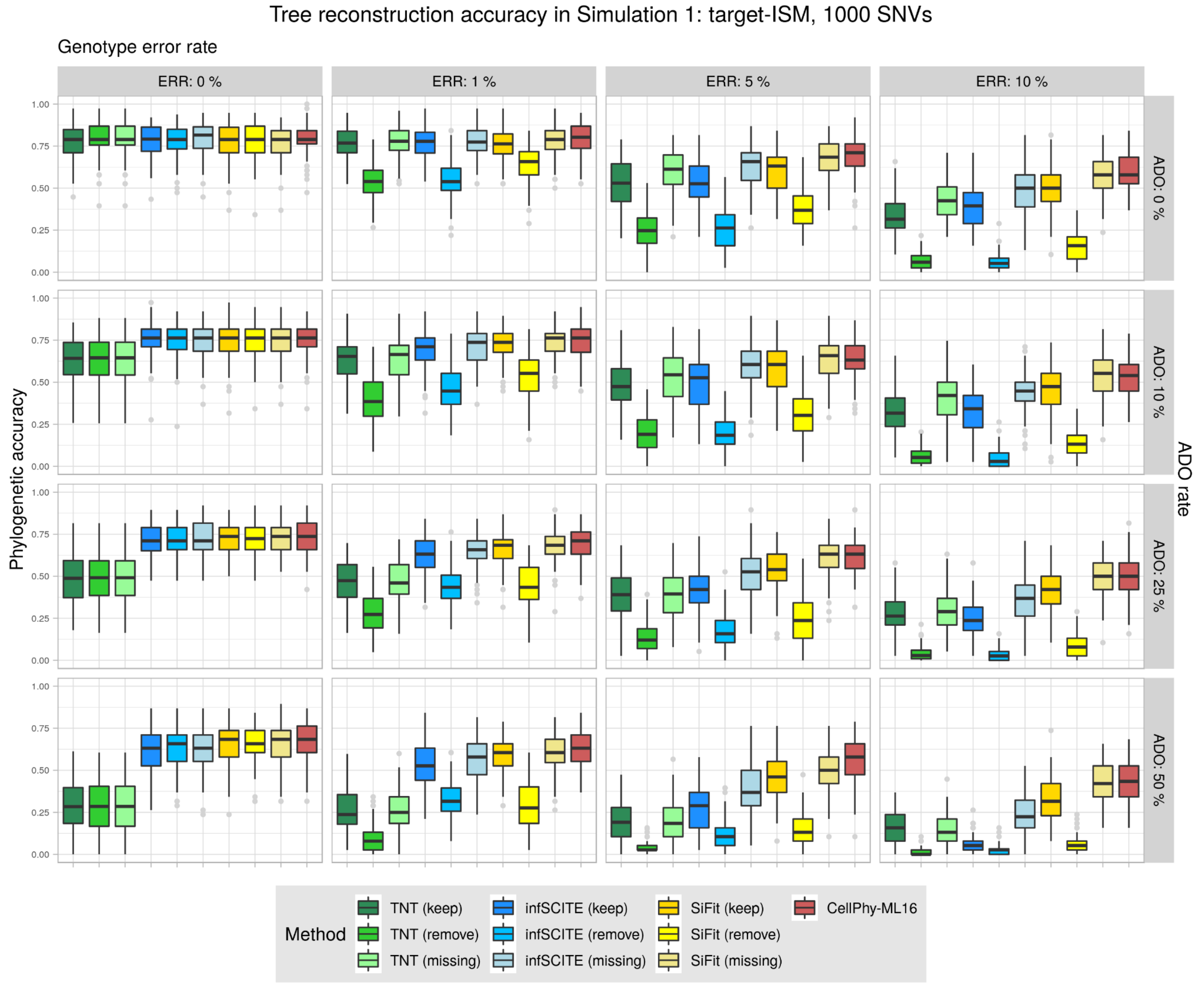

Fig. S4. Phylogenetic accuracy in Simulation 1 (“target-ISM”) with 1,000 SNVs. Datasets consisted of 40 cells. Accuracy was evaluated under different levels of genotype error (ERR), allelic dropout (ADO), and genotype recoding strategies (“keep”, “remove”, “missing”) as explained in the main text. Phylogenetic accuracy is defined as 1 – nRF (see Methods). See Fig. S2 for an explanation of the boxplots.

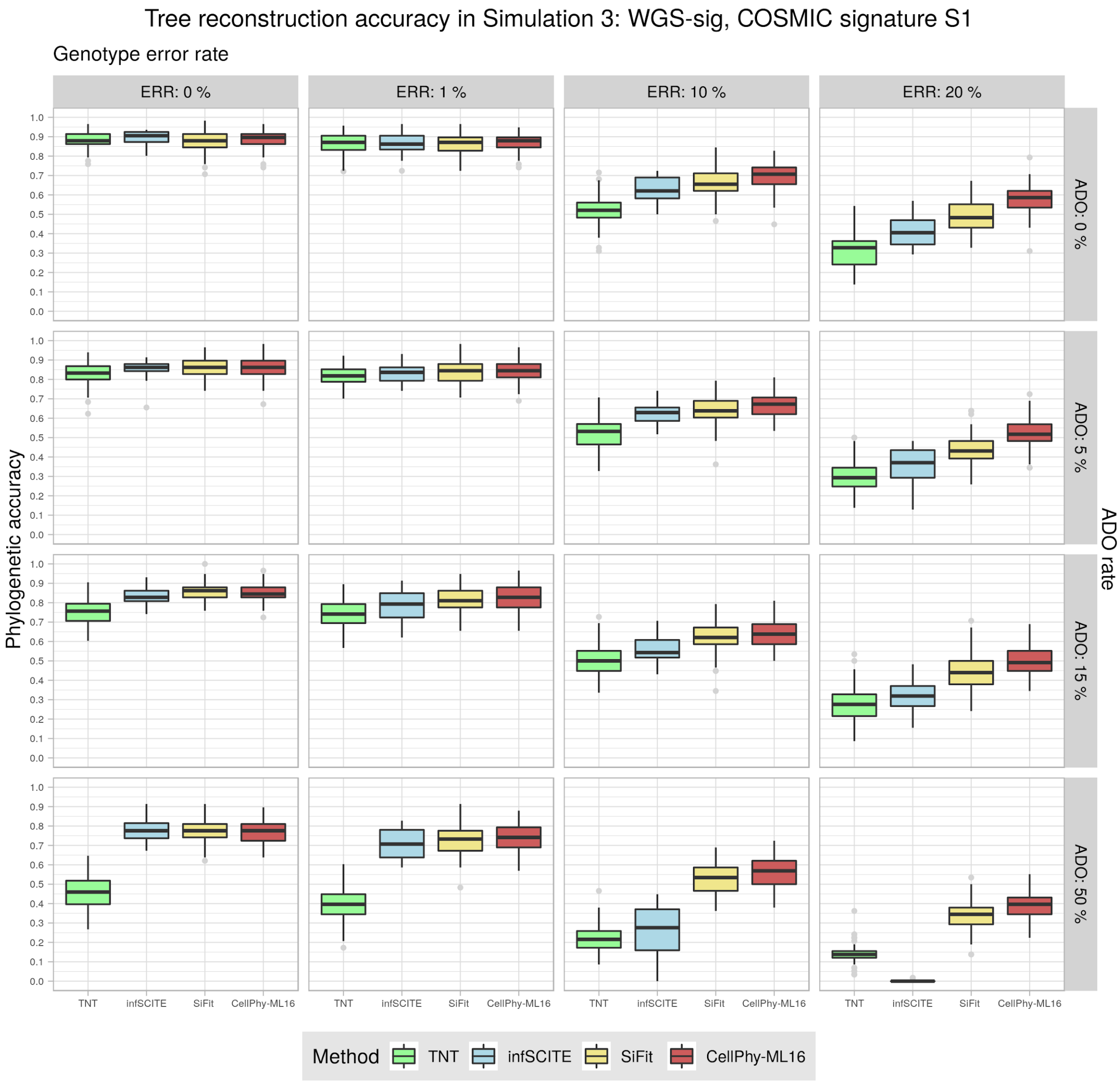

Fig. S5. Phylogenetic reconstruction accuracy in Simulation 3 ("WGS-sig") with signature S2. Datasets consisted of 60 cells and 1000-4000 SNVs. Accuracy was evaluated under different levels of genotype error (ERR) and allelic dropout (ADO). Phylogenetic accuracy is defined as 1 – nRF (see Methods). See Fig. S2 for an explanation of the boxplots.

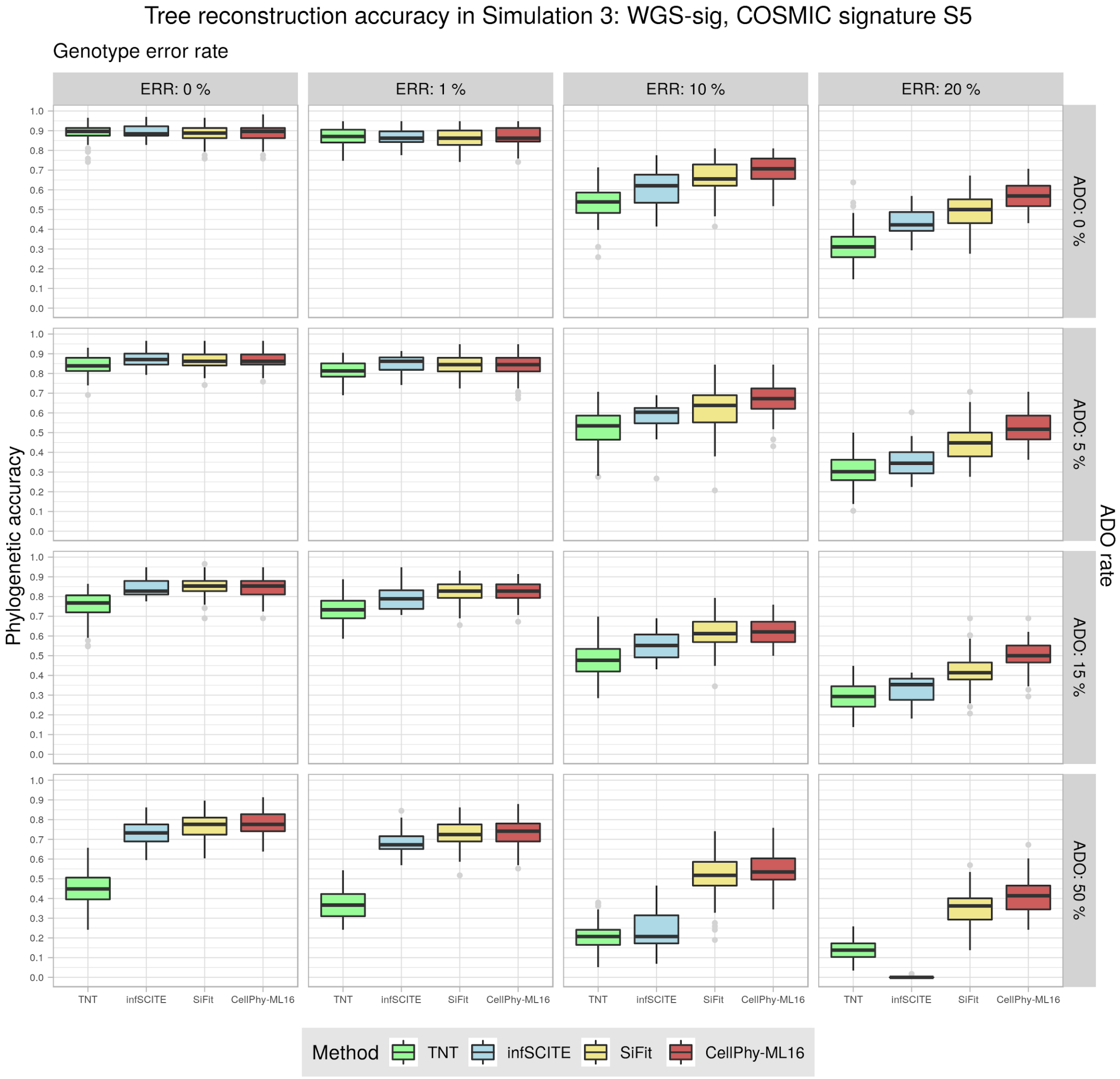

Fig. S6. Phylogenetic reconstruction accuracy in Simulation 3 ("WGS-sig") with signature S6. Datasets consisted of 60 cells and ~1000-4000 SNVs. Accuracy was evaluated under different levels of genotype error (ERR) and allelic dropout (ADO). Phylogenetic accuracy is defined as 1 – nRF (see Methods). See Fig. S2 for an explanation of the boxplots.

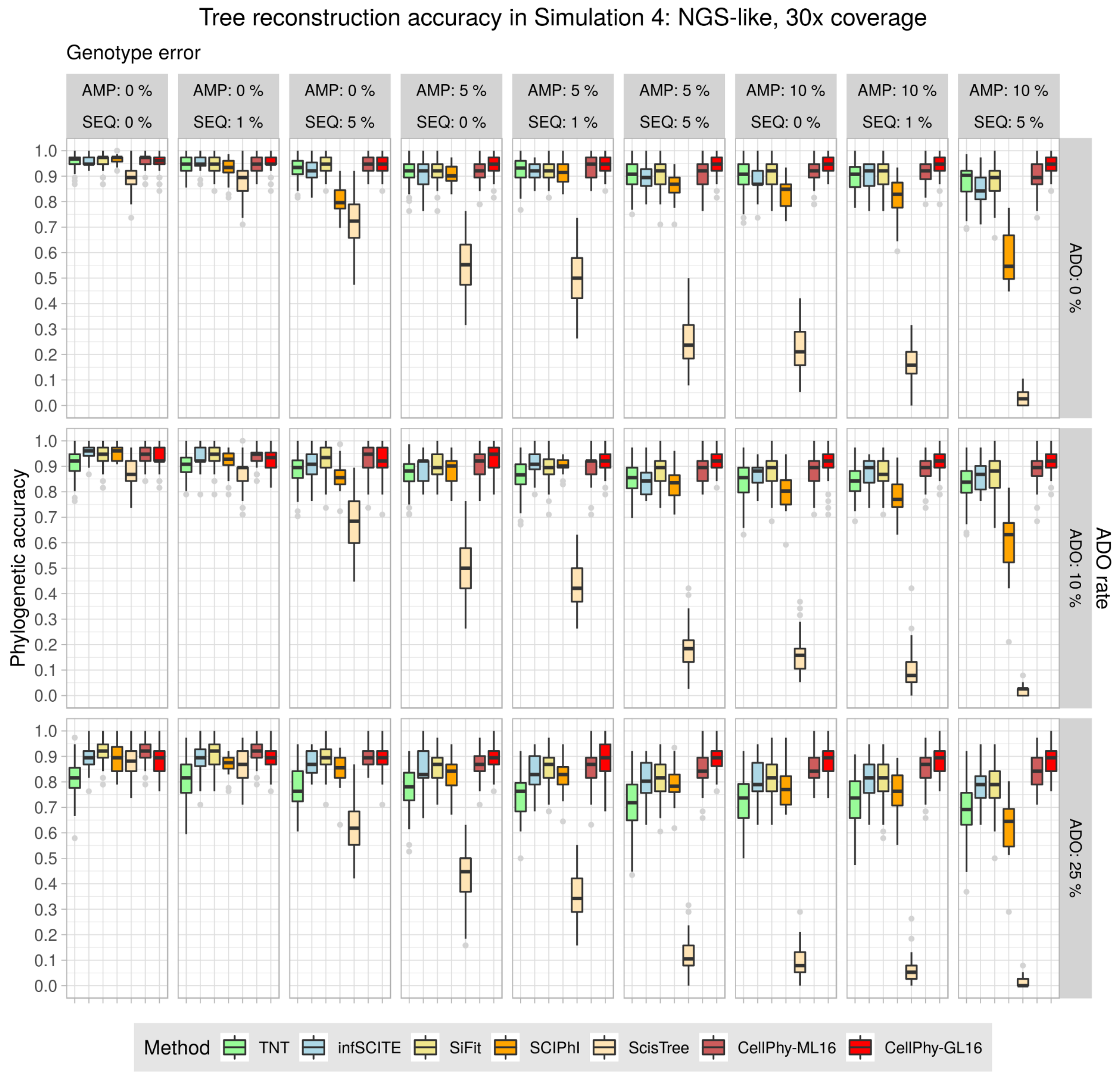

Fig. S7. **Phylogenetic accuracy in Simulation 4 ("NGS-like") at 30x**. Datasets consisted of 40 cells and ~1000-2000 SNVs. All methods use the ML genotypes except CellPhy-GL, which uses the genotype likelihoods. Phylogenetic accuracy is defined as 1 – nRF (see Methods). AMP is the amplification error rate, SEQ is the sequencing error rate, and ADO is the allelic dropout rate. See Fig. S2 for an explanation of the boxplots.

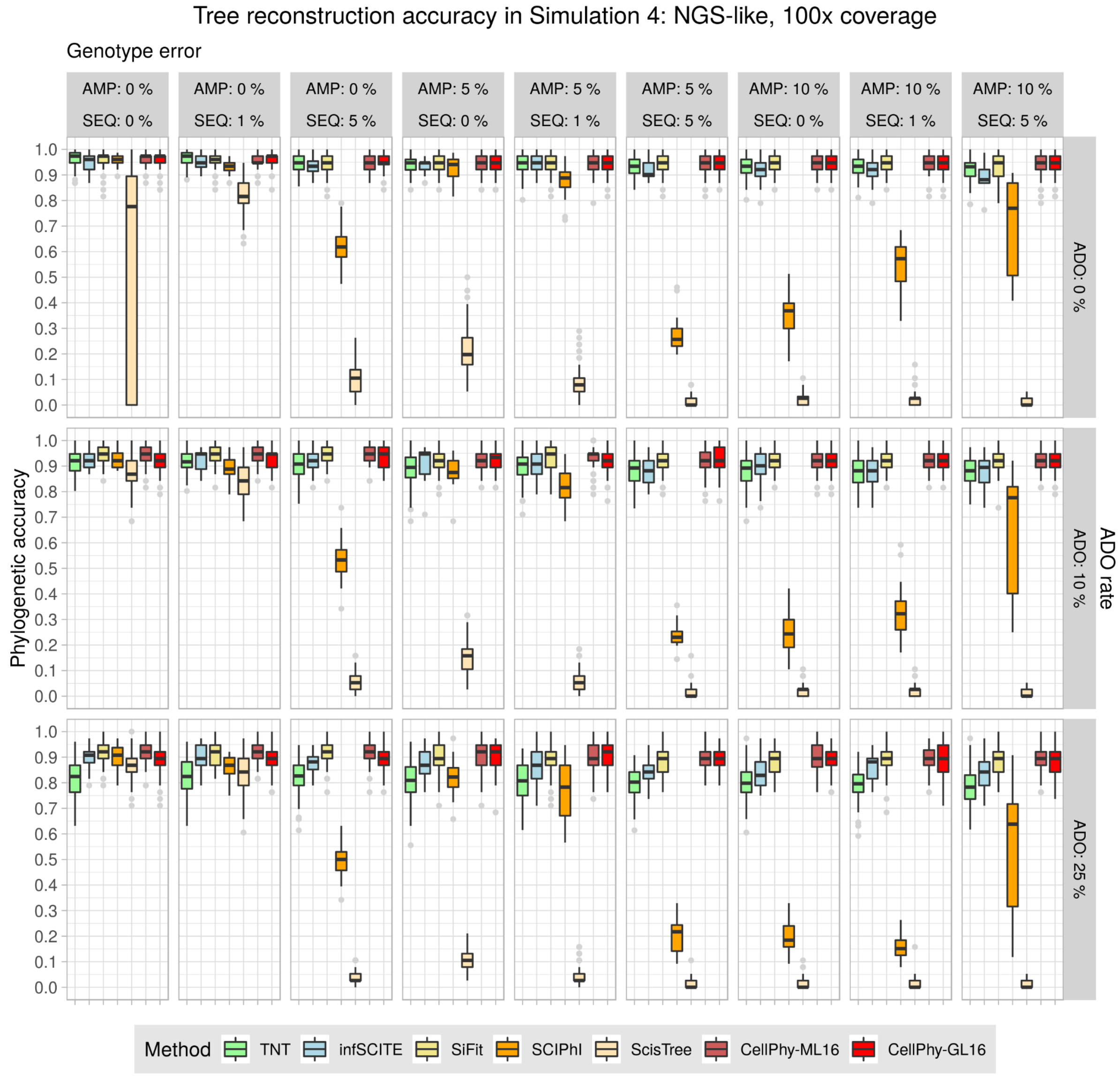

Fig. S8. Phylogenetic accuracy in Simulation 4 ("NGS-like") at 100x. Datasets consisted of 40 cells and ~1000-2000 SNVs. All methods use the ML genotypes except CellPhy-GL, which uses the genotype likelihoods. Phylogenetic accuracy is defined as 1 – nRF (see Methods). AMP is the amplification error rate, SEQ is the sequencing error rate, and ADO is the allelic dropout rate. See Fig. S2 for an explanation of the boxplots.

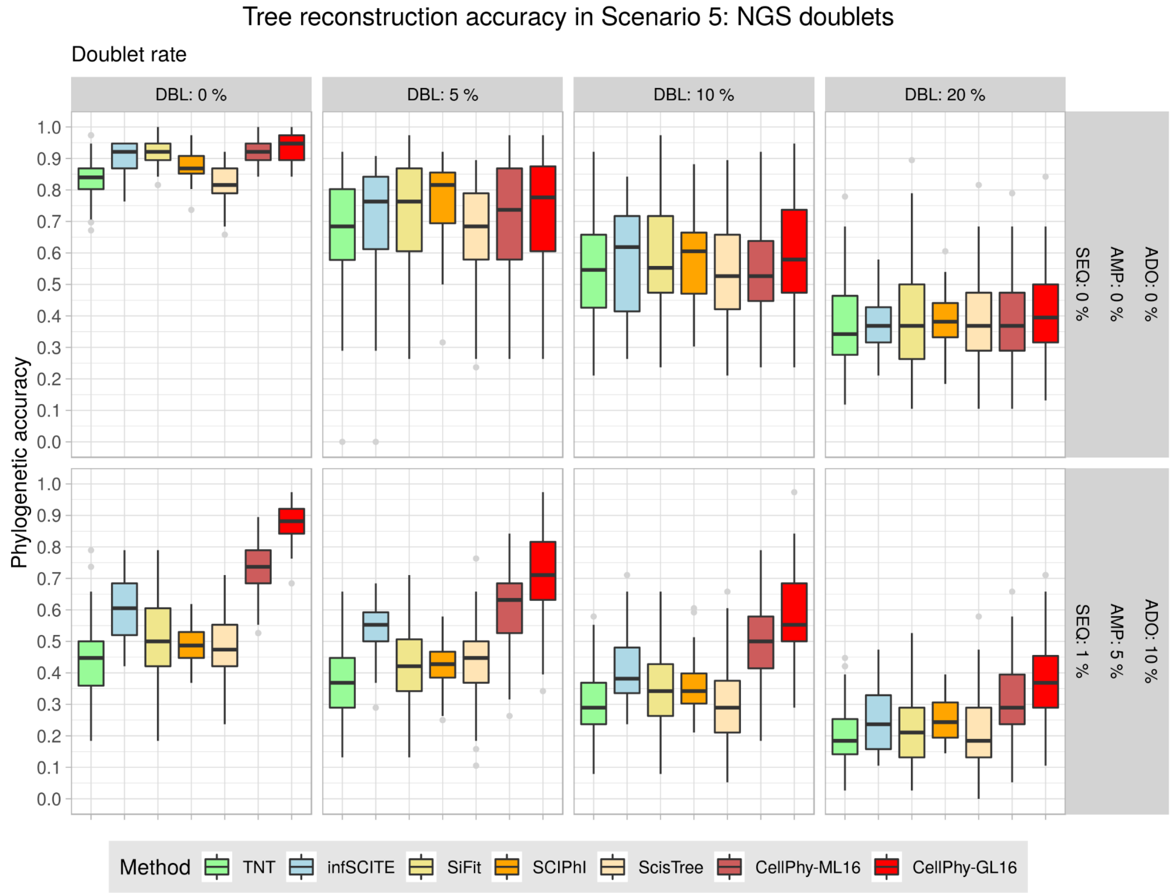

Fig. S9. Phylogenetic accuracy in Simulation 5 ("NGS-doublet"). Data simulated under mutational signature S2 and with a 5x sequencing depth. All methods use the ML genotypes except CellPhy-GL, which uses the genotype likelihoods. Phylogenetic accuracy is defined as 1 – nRF (see Methods). DBL is the doublet rate, AMP is the amplification error rate, SEQ is the sequencing error rate, and ADO is the allelic dropout rate. See Fig. S2 for an explanation of the boxplots.

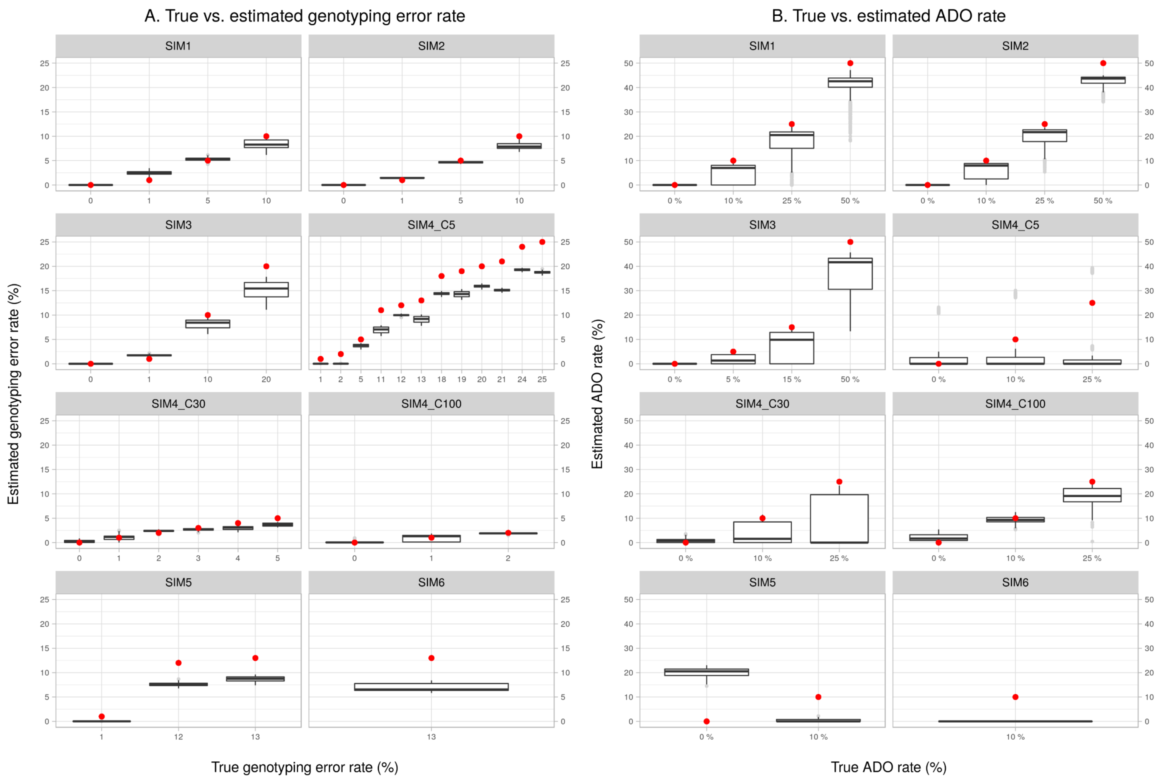

Fig. S10. Estimation of the genotype error and ADO rate. A Genotype error ML estimates. **B** ADO rate ML estimates. For simulation 1, 250, 500 or 1,000 SNVs were considered. Red dots highlight the true values. See Fig. S2 for an explanation of the boxplots.

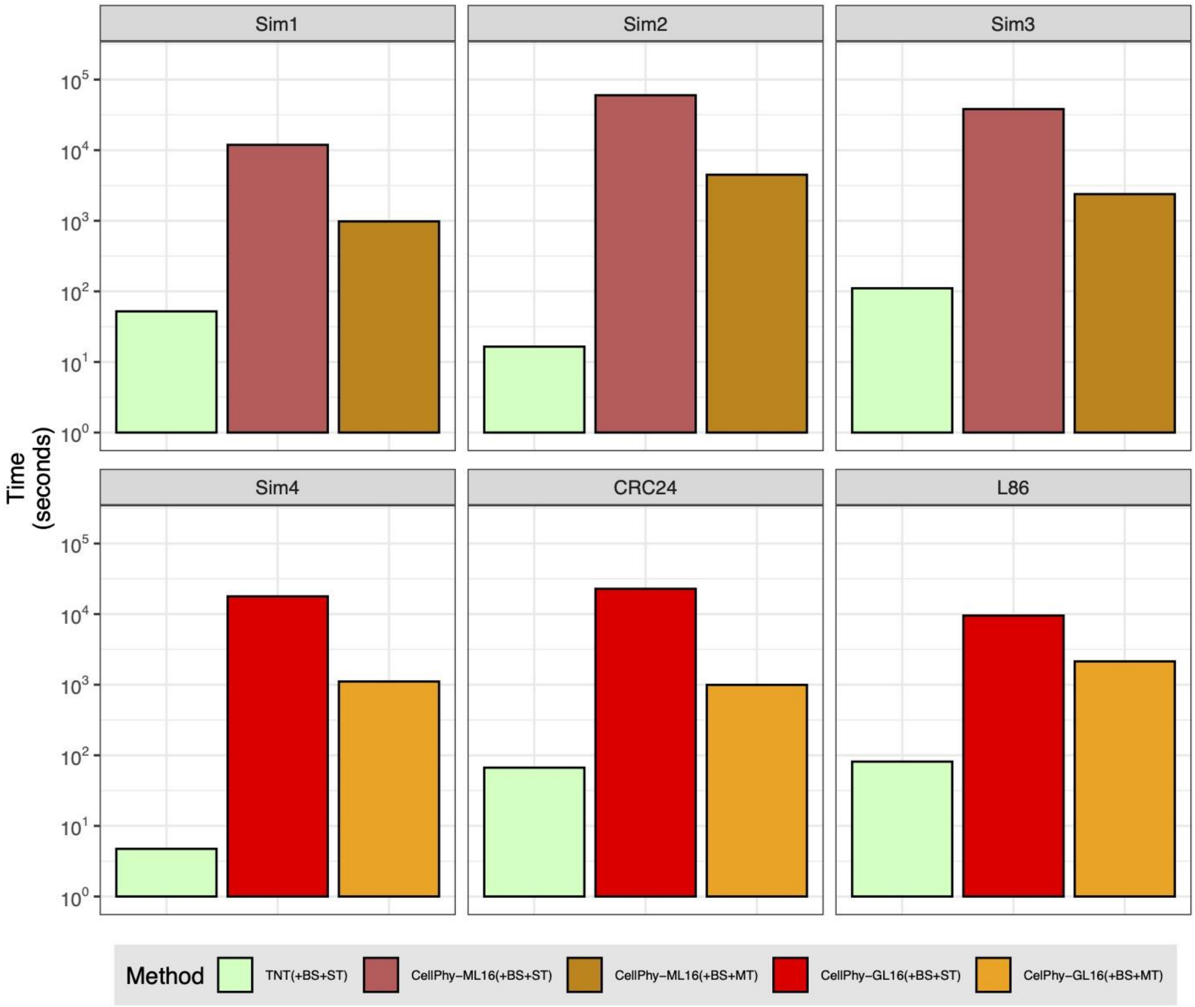

Fig. S11. Time-to-completion with 100 bootstrap replicates using single and multi-threading. Sim1–Sim4 are simulated datasets with 30, 100, 60, and 40 cells and 4753, 9935, 9982, and 3986 SNVs, respectively. CRC24 and L86 correspond to two of the empirical datasets described in the main text. Note the logarithmic time scale on the y-axis. CellPhy-ML16: ML genotype error model; CellPhy-GL16: genotype likelihood model; +BS: 100 bootstrap replicates; +ST = single-threading; +MT = multi-threading (24 cores).

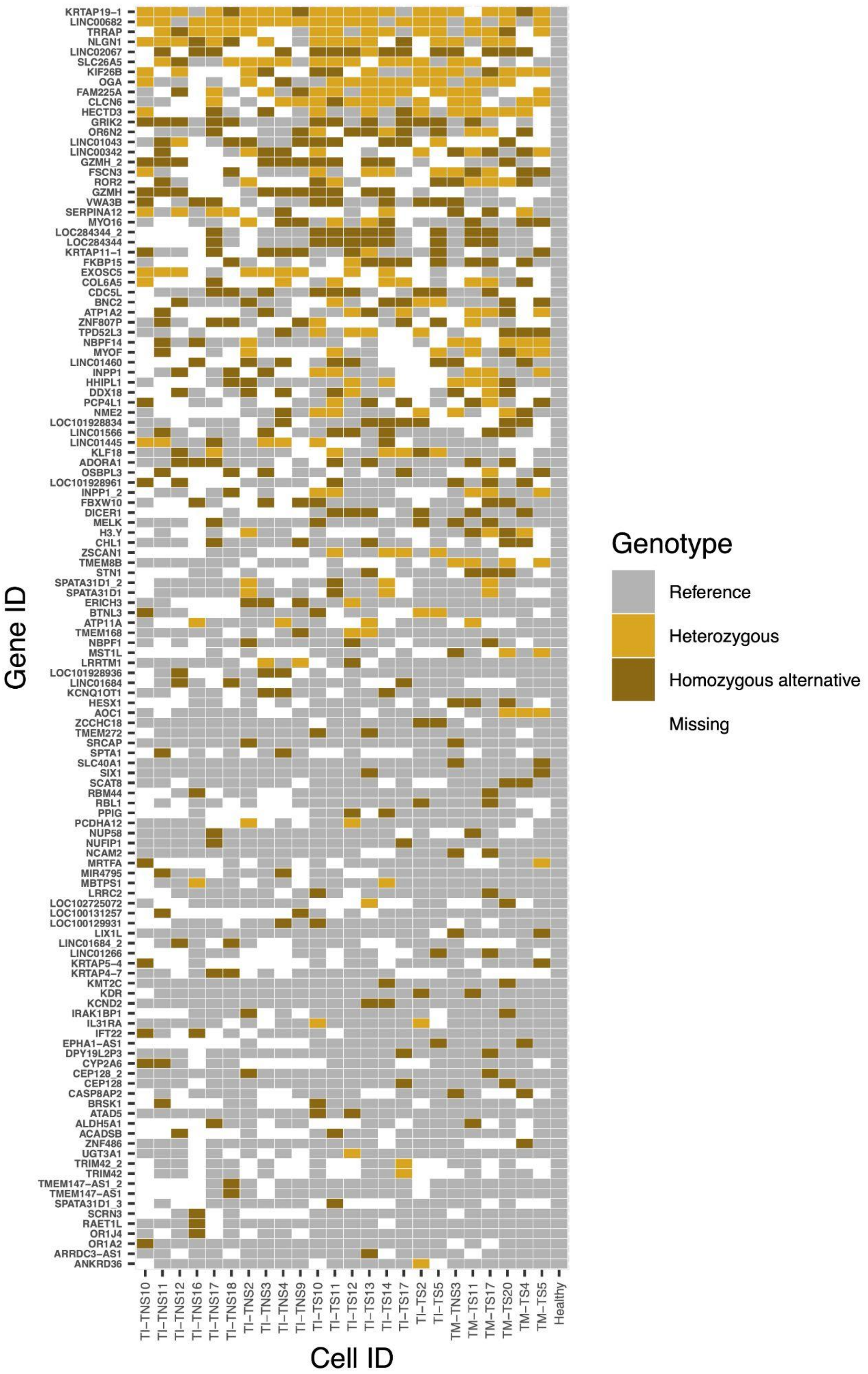

Fig. S12. CRC24 genotype matrix for exonic variants. Genotype matrix of somatic mutations identified in the CRC24 single-cell dataset (grey=reference homozygous, light gold=heterozygous, dark gold=alternative homozygous, white=missing). Only exonic mutations are shown (n=126), sorted according to their prevalence across the cell population. Gene names are displayed at the left of the map. Each column represents an individual cell.

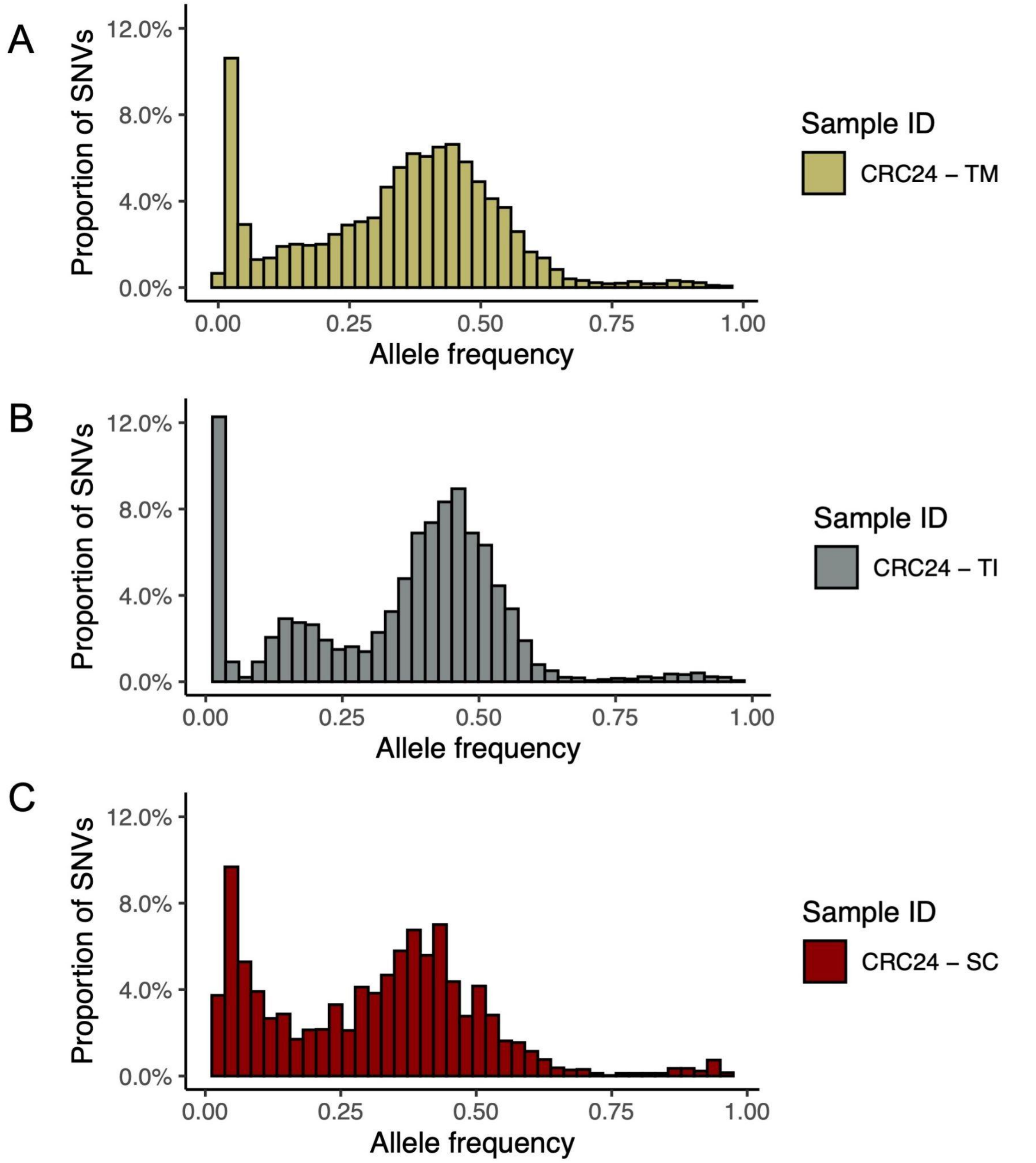

Fig. S13. CRC24 variant allele frequency distribution in single-cell and bulk data. A Tumor middle - TM (bulk sample) variant allele frequency (VAF) spectrum obtained from read depth of alternative alleles. **B** Tumor inferior - TI (bulk sample) VAF spectrum obtained from read depth of alternative alleles. **C** Single-cell - SC VAF spectrum obtained from allele frequency of genotype calls. All VAF distributions were derived from 3,936 shared SNVs between the single-cells and the bulk samples.

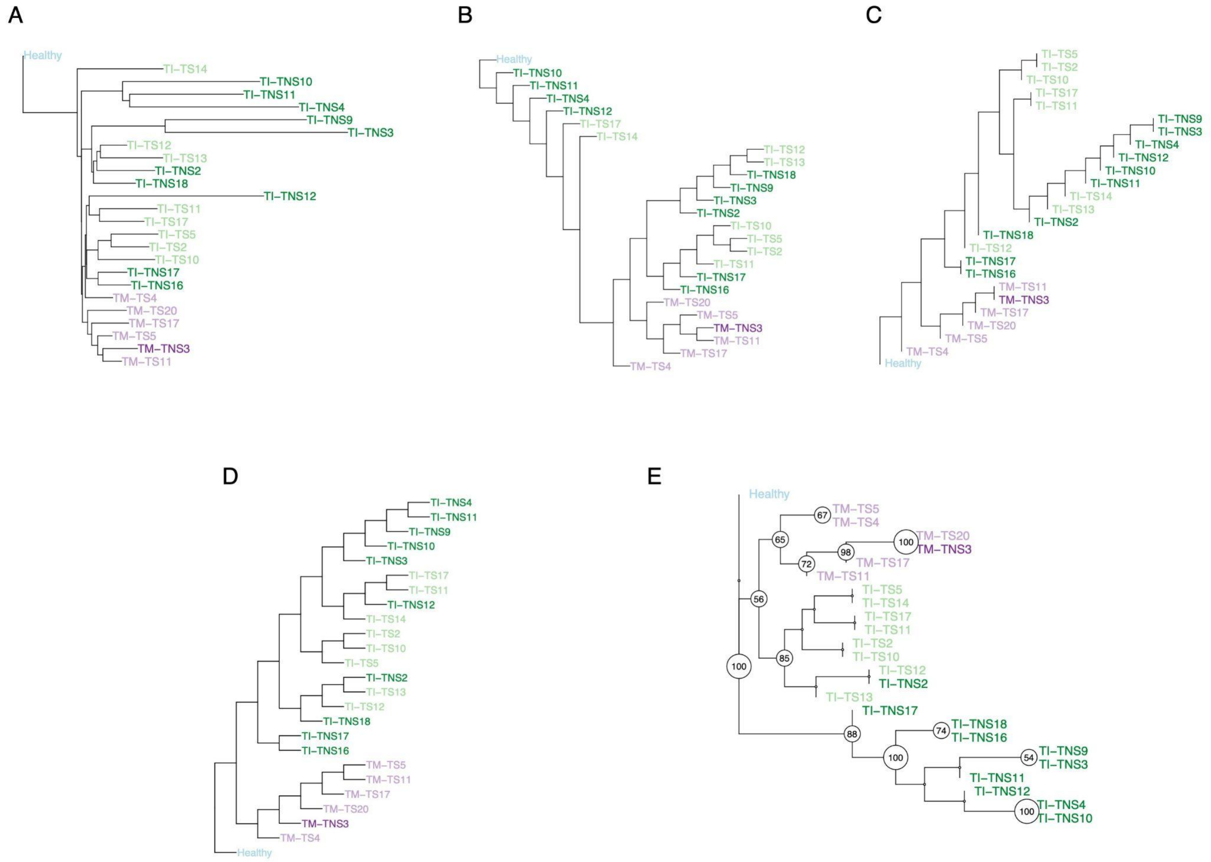

Fig. S14. SiFIT, SCIPhI, infSCITE, ScisTree, and TNT trees for the CRC24 dataset. A SiFit tree. **B** SCIPhI tree. **C** infSCITE tree. **D** ScisTree tree. **E.** TNT tree (only bootstrap values above 50 are shown)**.** Distinct colors represent cell type: healthy (blue); tumor-non-stem from TI region (dark green), tumor-stem from TI region (light green), tumor-non-stem from TM region (dark purple), tumor-stem from TM region (light purple).

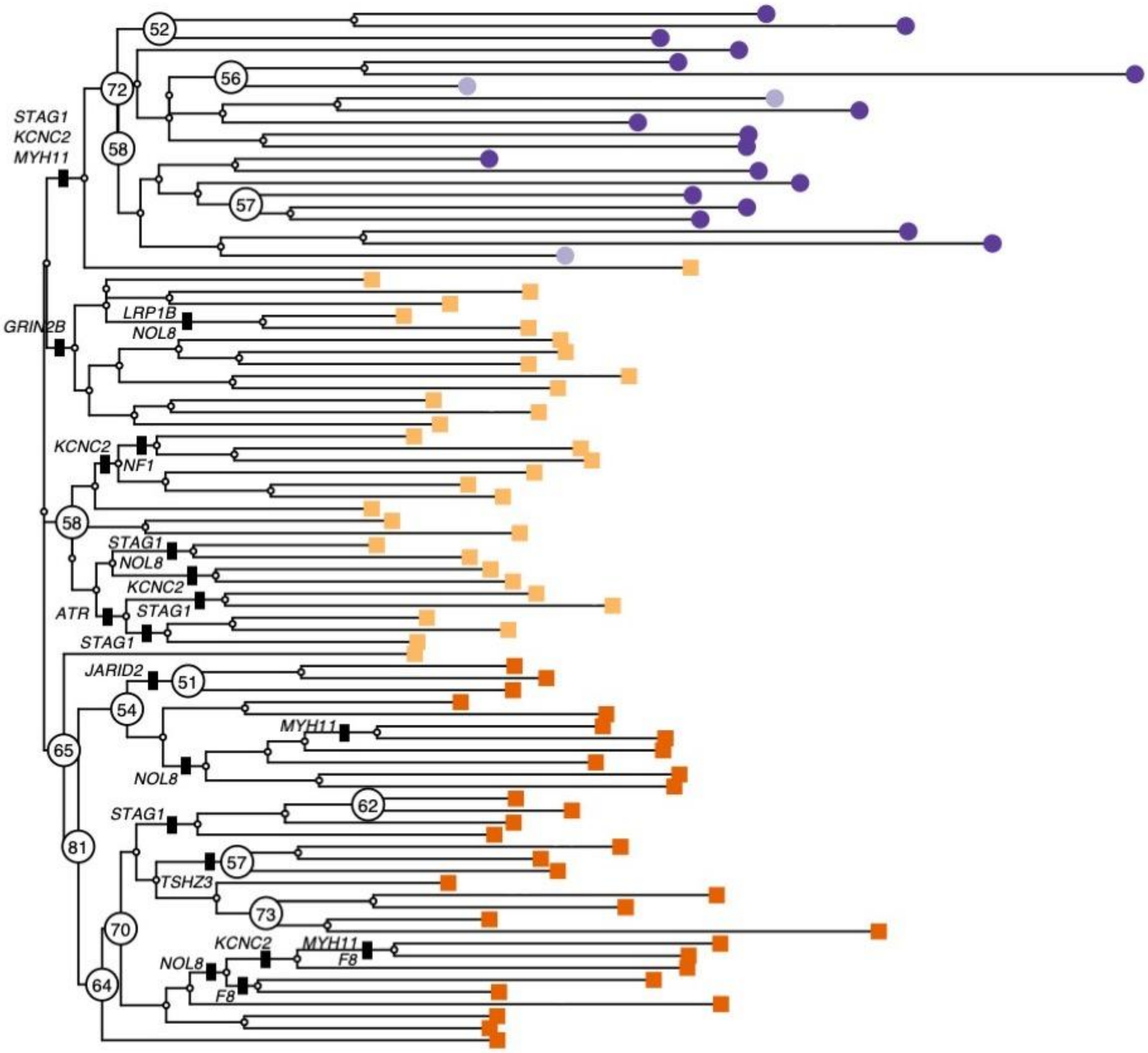

Fig. S15. Non-synonymous mutations along the CellPhy L86 tree. The dataset analyzed is a subset of the data for patient CRC2 in [21]. The figure shows the CellPhy L86 tree with non-synonymous mutations mapped to the internal branches of the tree. Distinct shapes and colors represent cell type: healthy diploid cells - from both primary and metastatic sites - (dark purple circle), healthy diploid cells missorted (light purple circle), primary tumor aneuploid cells (light orange square), metastatic aneuploid cells (dark orange square). Only bootstrap values above 50 are shown.

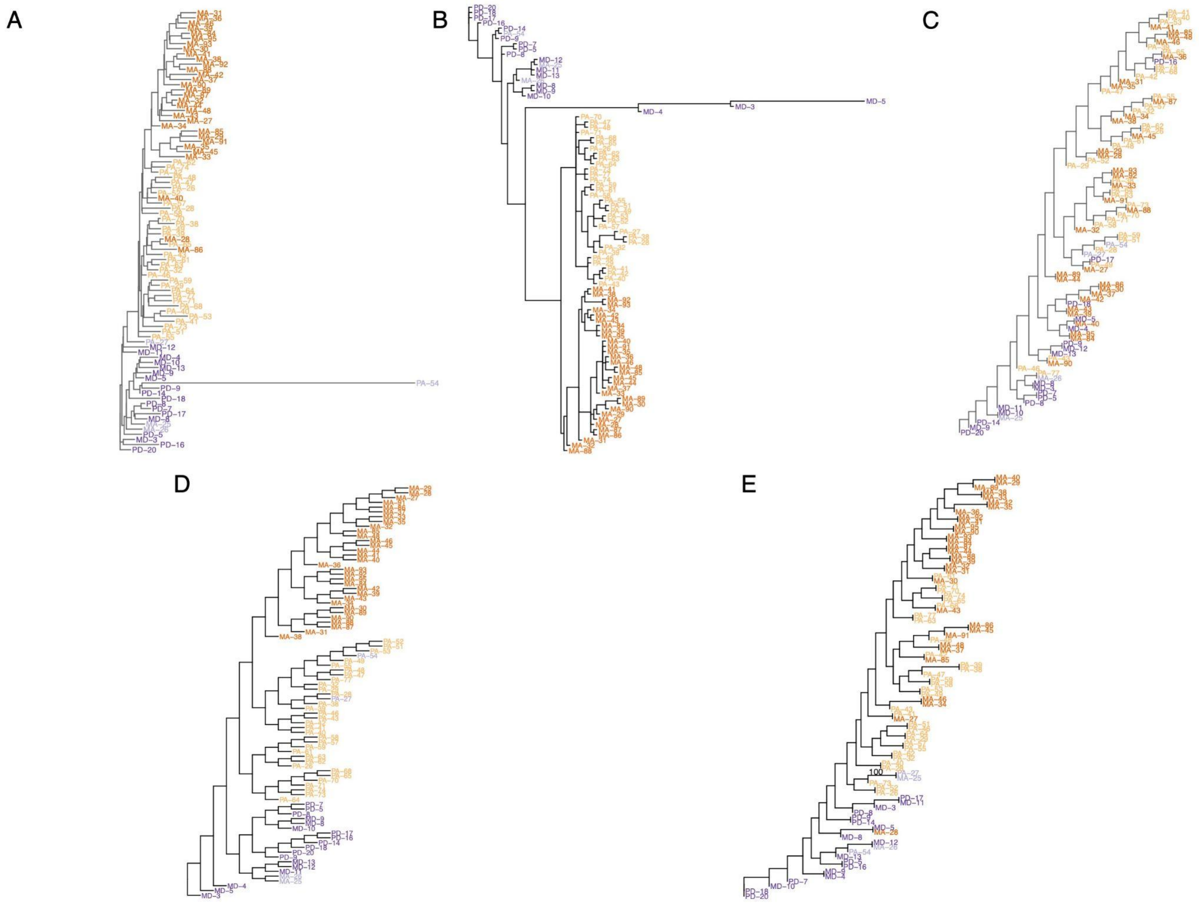

Fig. S16. SiFiT, SCIPhI, infSCITE, ScisTree, and TNT trees for the L86 dataset. The dataset analyzed is a subset of the data for patient CRC2 in [21]. **A** SiFit tree. **B** SCIPhI tree. **C** infSCITE tree. **D** ScisTree tree. **E.** TNT tree (only bootstrap values above 50 are shown). Distinct colors represent cell type: healthy diploid cells from both primary and metastatic sites (dark purple), healthy diploid cells missorted (light purple), primary tumor aneuploid cells (light orange), metastatic aneuploid cells (dark orange).

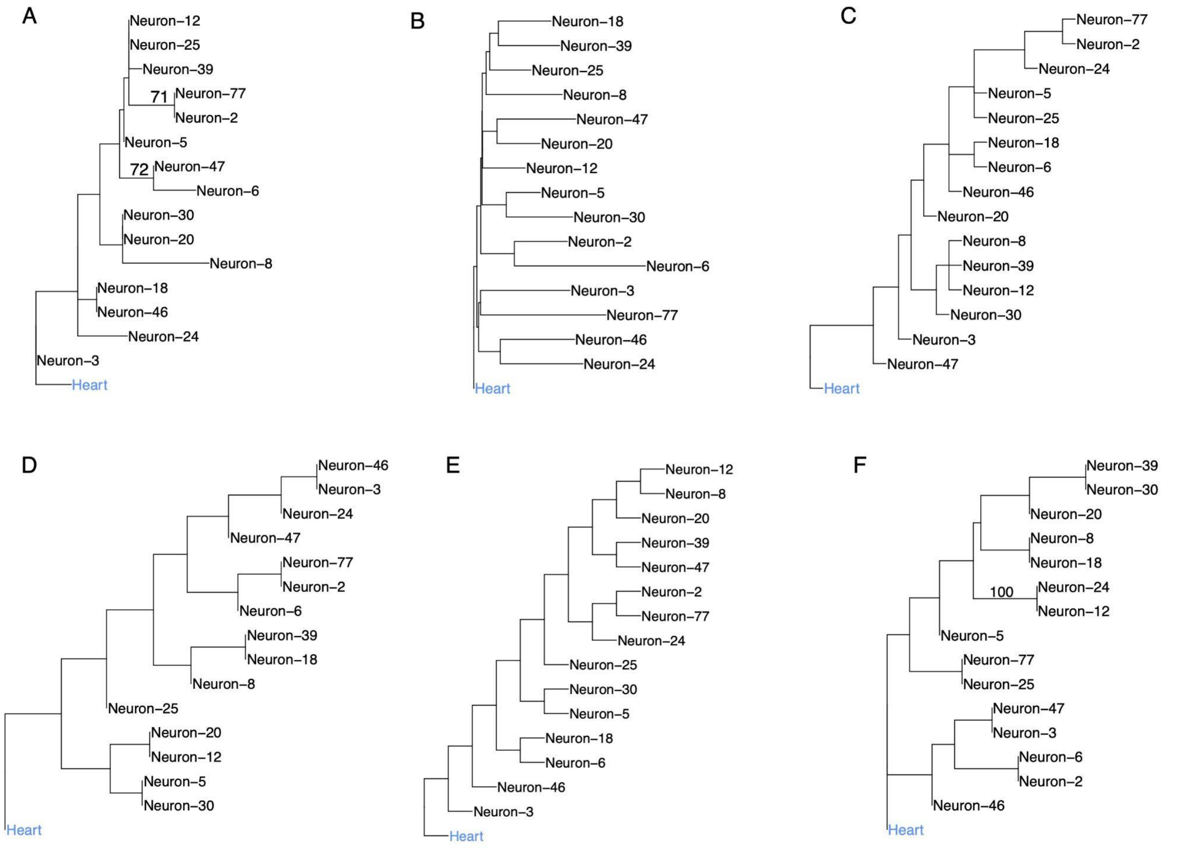

Fig. S17. Phylogenetic reconstruction from single neurons. Data consisted of 15 whole-genome sequenced neurons from a healthy donor [69]. **A** Single-cell tree inferred with “Cellphy-GL”. **B** SiFit tree. **C** SCIPhI tree. **D** infSCITE. **E** ScisTree. **F** TNT tree. In A and F, only bootstrap values above 50 are shown.

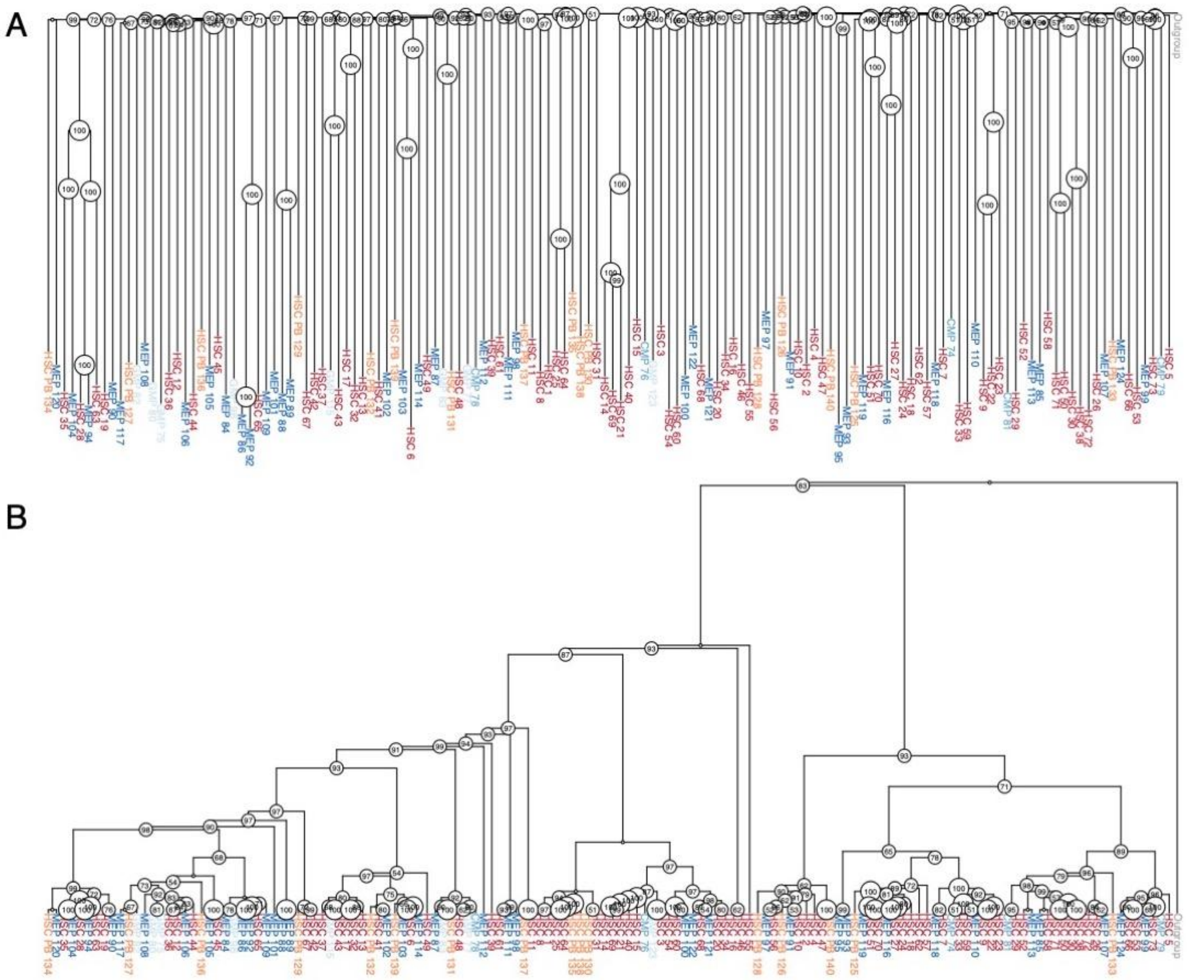

Fig. S18. Phylogenetic reconstruction from single-cell hematopoietic colonies. Data consists of 140 whole-genome sequenced single-cell derived hematopoietic “colonies” from a healthy donor [15]. **A** ML phylogenetic tree inferred with Cellphy using a genotype matrix with 127,884 mutations. Only bootstrap support values above 50 are shown. **B** The same ML phylogeny as in (A), but ignoring branch lengths to ease visualization of ancestral relationships. Only bootstrap values above 50 are shown.

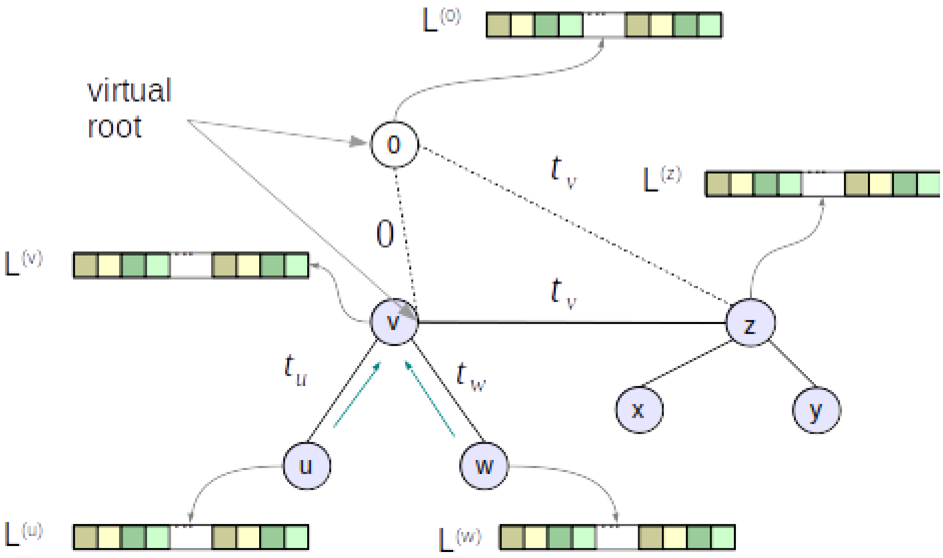

Fig. S19. Genotype likelihood vectors for a simple unrooted phylogenetic tree. To compute the likelihood of the tree via the Felsenstein pruning algorithm, we place a virtual root (*node 0*) at an arbitrary inner node (here: *node v*). Then, we perform a post-order tree traversal and compute likelihood vectors at each inner node recursively according to Equation (19). We use vectors L^u and L^w to compute L^v, vectors L^x and L^y (not shown) to compute L^z, and then finally vectors L^v and L^z to compute L^0.

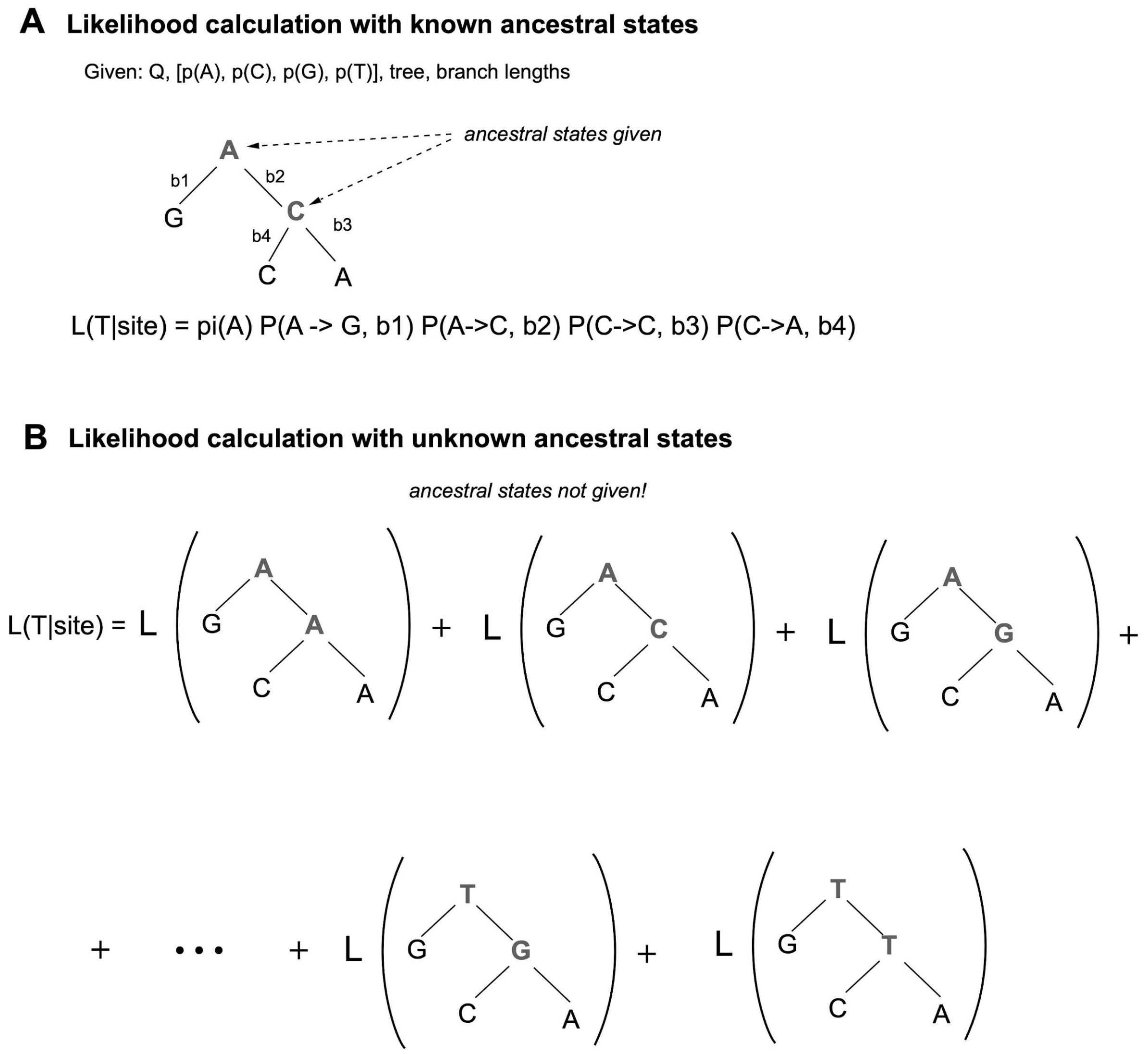

Fig. S20. Phylogenetic likelihood calculations. A Outline of a simple likelihood calculation on a given tree with given branch lengths, when the inner states are also given. **B** The likelihood is just the sum over the likelihoods of *all possible* evolutionary scenarios. The likelihood of the trees in parentheses is calculated as in the simple example for given states.

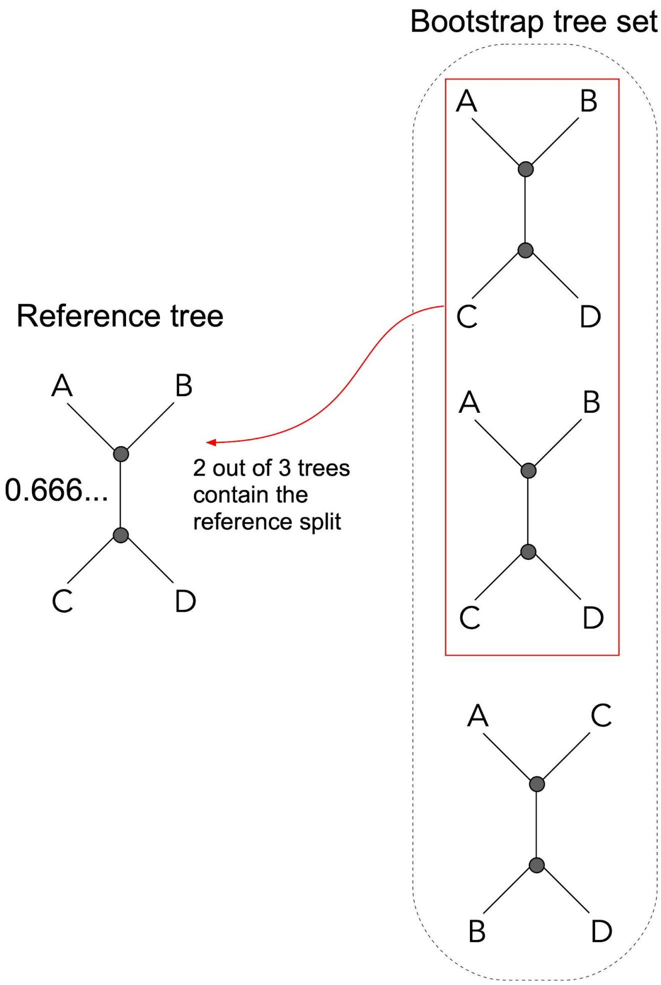

Fig. S21. Phylogenetic bootstrap calculation. Outline of bootstrap proportion/branch support computations. For each inner branch in the reference tree on the left, we count how frequently the bipartition/split (*AB|CD* in the example) occurs in the set of bootstrap trees on the right. Here the bipartition/split/branch is present in two out of three bootstrap trees, and hence the branch support is ⅔.

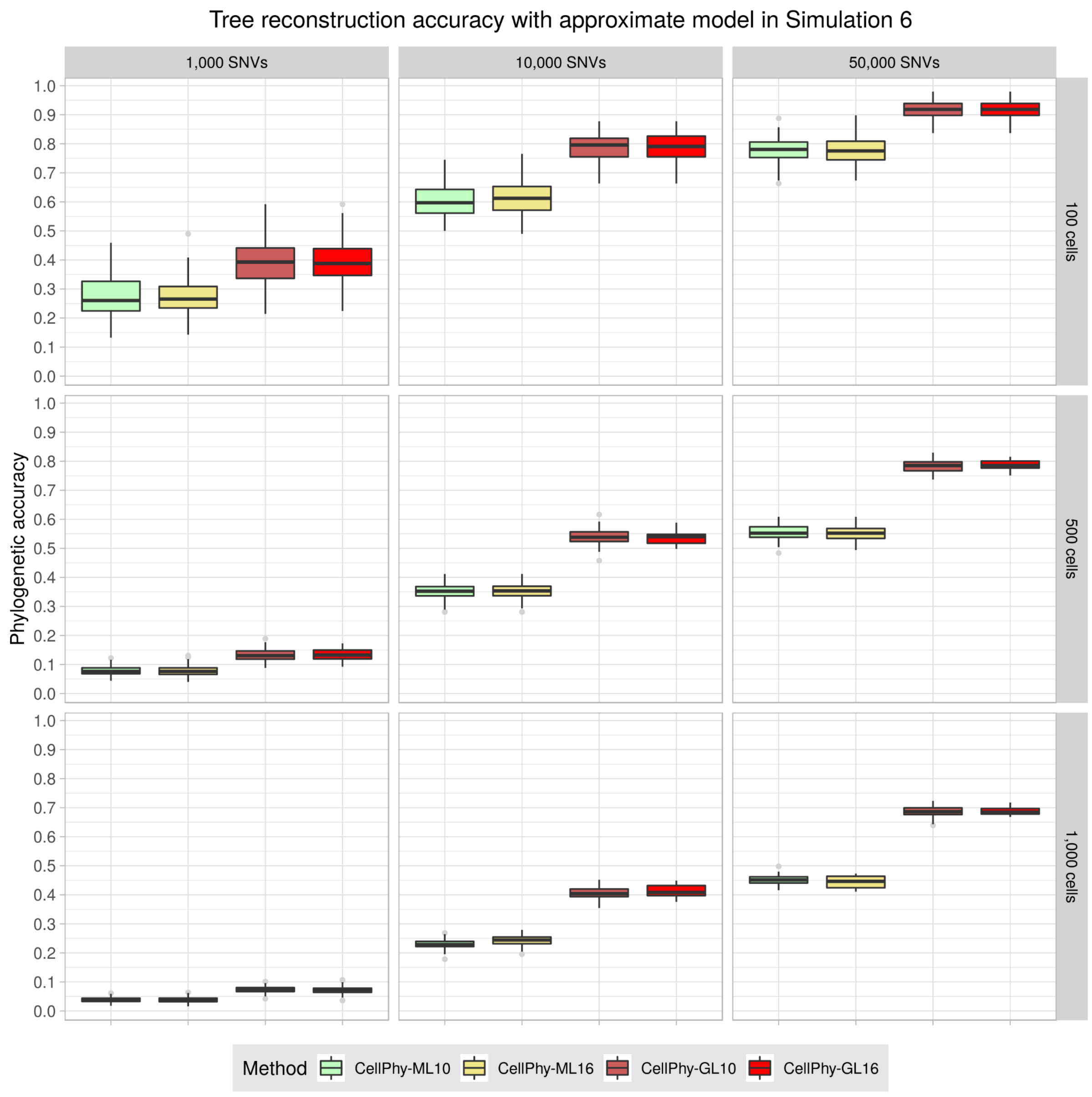

Fig. S22. Phylogenetic accuracy with approximate model GT10 in Simulation 6 ("NGS-large"). The approximate 10-state model for unphased genotypes (ML10/GL10) yields very similar accuracy to the original 16-state model (ML16/GL16). This observation holds for all simulations in our study (data not shown). Data simulated under mutational signature S2 and with a 5x sequencing depth. Phylogenetic accuracy is defined as 1 – nRF (see Methods). See Fig. S2 for an explanation of the boxplots.

Supplementary Note 1. Genotype error model

P (N | M) is the probability of observing the single-cell genotype N after sequencing, given the true genotype M, diploid and biallelic. We consider two types of technical errors resulting in a wrong genotype, allelic dropout (ADO) and amplification/sequencing error (ERR), which occur at rates δ and ε, respectively. Note that we allow for the presence of both ADO and ERR in the same observed genotype.

Allelic dropout occurs during single-cell whole-genome amplification (scWGA) when one of the two alleles is not amplified and cannot be represented in the observed data. ADO implies a single allele, as otherwise the genotype is "missing". Thus, the rate δ is the probability that the amplification of one or the other allele has failed, and therefore that we observe the homozygous genotype defined by the amplified allele. Given the phased genotype a|b, and with "_ " indicating the dropped allele:

$$\text{ADO rate } (\delta) = P(\_|b \mid a|b) + P(a|\_ \mid a|b)$$

An amplification/sequencing error occurs when the observed allele is not the true allele. Given that the ERR rate tends to be small, we assume a maximum of one ERR per genotype. Specifically:

$$\text{ERR rate } (\varepsilon) = P(b|a), \text{ where } b \mathrel{!=} a$$

Phased genotypes error model

Under these assumptions, there are seven scenarios (I-VII) with non-zero probability for the calculation of P (N | M) for phased genotypes, with alleles *a-d*:

If the true genotype is homozygous

I. $P(a|a \mid a|a) = (1 - \delta) * (1 - \varepsilon) + \delta * (1 - \frac{1}{2}\varepsilon) = 1 - \varepsilon + \frac{1}{2}\delta\varepsilon$

For example, P (AA | AA). This can happen in two ways: (1) without ADO (i.e., $1 - \delta$) and without ERR (i.e., $1 - \varepsilon$), or (2) after ADO in either allele (δ) and without ERR in the non-dropped allele (i.e., $1 - \frac{1}{2}\varepsilon$).

II. $P(a|b \mid aa) = (1 - \delta) * \frac{1}{2}\frac{1}{3}\varepsilon = (1 - \delta) * \frac{1}{6}\varepsilon$

For example, P (AT | AA). This can only happen without ADO (i.e., $1 - \delta$), followed by an ERR in a specific allele that is converted to one of the other three nucleotides (i.e., $\frac{1}{2}\frac{1}{3}\varepsilon$).

III. $P(b|b \mid a|a) = \delta * \frac{1}{2}\frac{1}{3}\varepsilon = \frac{1}{6}\delta\varepsilon$

For example, P (TT | AA). Because we ignore more than one ERR per genotype, this can only happen through ADO in either allele (i.e., δ), followed by an ERR in the non-dropped allele (i.e., $\frac{1}{2}\frac{1}{3}\varepsilon$).

For a given true homozygous genotype, we have 1 I, 6 II, 3 III combinations, so the sum of probabilities is: $1 - \varepsilon + \frac{1}{2}\delta\varepsilon + \varepsilon - \delta\varepsilon + \frac{1}{2}\delta\varepsilon = 1$

If the true genotype is heterozygous

IV. P (*a*|*a* | *a*|*b*) = $(1 – δ) * ½ ⅓ ε + ½ δ * (1 – ½ ε) + ½ δ * ½ ⅓ ε = ½ δ + ⅙ ε – ⅓ δε$

For example, P (AA | AT). This can happen in three ways: (1) without ADO (i.e., 1 – δ) and with ERR in allele j (i.e.,½ ⅓ ε), (2) after ADO in allele j (i.e., ½ δ) followed by no ERR in allele i (i.e., 1 – ½ ε), or (3) after ADO in allele i (i.e., ½ δ) followed by an ERR in allele j (i.e., ½ ⅓ ε).

V. P (*c*|*c* | *a*|*b*) = $δ * ½ ⅓ ε = ⅙ δε$

For example, P (CC | AT). Because we ignore the possibility of two ERR in the same genotype, this can only occur if there is ADO in either allele (i.e., δ), followed by an ERR in the non-dropped allele (i.e., ½ ⅓ ε).

VI. P (*a*|*c* | *a*|*b*) = $(1 – δ) * (½ ⅓ ε) = (1 – δ) ⅙ ε$

For example, P (AC | AT). This can only happen without ADO (i.e., 1 – δ), through an ERR in allele j (i.e., ½ ⅓ ε).

VII. P (*a*|*b* | *a*|*b*) = $(1 – δ) * (1 – ε)$

For example, P (AT | AT). This case can only happen without ADO (i.e., 1 – δ) and without ERR (i.e., 1 – ε).

For a given true heterozygous genotype, we have 2 IV, 2 V, 4 VI, and 1 VII combinations, so the sum of probabilities is $δ + ⅓ ε – ⅔ δε + ⅓ δε + (1 – δ) ⅔ ε + (1 – δ) * (1 – ε) = 1$. Given the model's assumptions, P (*N* | *M*) is zero for the remaining scenarios.

Unphased genotypes error model

For unphased genotypes, the error model is identical to the phased case, except for scenario II:

P (*a*|*b* | *a*|*a)* = $(1 – δ) * ½ ⅓ ε = (1 – δ) * ⅓ ε$

For example, P (AT | AA). This can only happen without ADO (i.e., 1 – δ), followed by an ERR in one of the alleles converted to one of the other three nucleotides (i.e., ⅓ ε).

Supplementary Note 2. Approximate model of evolution for unphased diploid genotypes with ten states

Current techniques for producing scDNA-seq data do not reveal the phase of the genotypes (i.e., we do not know which allele is located in the maternal or paternal chromosome). We have also implemented a specific model for unphased genotypes with only ten states that speed up the calculations. However, for unphased states, the probability of changing between a homozygous and a heterozygous genotype is not reversible, as, in principle, the change from genotype *aa* to genotype *ab* is twice more probable (either allele can change) than from *ab* to *aa* (change can occur only in allele *b*). Considering this asymmetry would result in a non-reversible Q matrix, which would yield the calculation of the tree likelihood much more complex and prohibitively slow, as it would require rooted trees. As a compromised, approximate solution, we implemented a GT10 model based on a reversible Q matrix (here called Q_{10}):

$$Q_{10} = \begin{array}{c} \\ A/A \\ C/C \\ G/G \\ T/T \\ A/C \\ A/G \\ A/T \\ C/G \\ C/T \\ G/T \end{array} \begin{array}{c} \begin{array}{cccccccccc} A/A & C/C & G/G & T/T & A/C & A/G & A/T & C/G & C/T & G/T \end{array} \\ \begin{pmatrix} -q_{A/A} & 0 & 0 & 0 & \alpha\pi_{A/C} & \beta\pi_{A/G} & \gamma\pi_{A/T} & 0 & 0 & 0 \\ 0 & -q_{C/C} & 0 & 0 & \alpha\pi_{A/C} & 0 & 0 & \kappa\pi_{C/G} & \lambda\pi_{C/T} & 0 \\ 0 & 0 & -q_{G/G} & 0 & 0 & \beta\pi_{A/G} & 0 & \kappa\pi_{C/G} & 0 & \mu\pi_{G/T} \\ 0 & 0 & 0 & -q_{T/T} & 0 & 0 & \gamma\pi_{A/T} & 0 & \lambda\pi_{C/T} & \mu\pi_{G/T} \\ \alpha\pi_{A/A} & \alpha\pi_{C/C} & 0 & 0 & -q_{A/C} & \kappa\pi_{A/G} & \lambda\pi_{A/T} & \beta\pi_{C/G} & \gamma\pi_{C/T} & 0 \\ \beta\pi_{A/A} & 0 & \beta\pi_{G/G} & 0 & \kappa\pi_{A/C} & -q_{A/G} & \mu\pi_{A/T} & \alpha\pi_{C/G} & 0 & \gamma\pi_{G/T} \\ \gamma\pi_{A/A} & 0 & 0 & \gamma\pi_{T/T} & \lambda\pi_{A/C} & \mu\pi_{A/G} & -q_{A/T} & 0 & \alpha\pi_{C/T} & \beta\pi_{G/T} \\ 0 & \kappa\pi_{C/C} & \kappa\pi_{G/G} & 0 & \beta\pi_{A/C} & \alpha\pi_{A/G} & 0 & -q_{C/G} & \mu\pi_{C/T} & \lambda\pi_{G/T} \\ 0 & \lambda\pi_{C/C} & 0 & \lambda\pi_{T/T} & \gamma\pi_{A/C} & 0 & \alpha\pi_{A/T} & \mu\pi_{C/G} & -q_{C/T} & \kappa\pi_{G/T} \\ 0 & 0 & \mu\pi_{G/G} & \mu\pi_{T/T} & 0 & \gamma\pi_{A/G} & \beta\pi_{A/T} & \lambda\pi_{C/G} & \kappa\pi_{C/T} & -q_{G/T} \end{pmatrix} \end{array}$$

In this case, we need to estimate five nucleotide exchangeabilities (α = r(*A*↔*C*), β = r(*A*↔*G*), γ= r(*A*↔*T*), κ = r(*C*↔*G*), λ = r(*C*↔*T*); *let* μ = r(*G*↔*T*) = *1*) and nine stationary unphased genotype frequencies ($\pi_{A/A}$, $\pi_{A/C}$, $\pi_{A/G}$, $\pi_{A/T}$, $\pi_{C/C}$, $\pi_{C/G}$, $\pi_{C/T}$, $\pi_{G/G}$, $\pi_{G/T}$; $\pi_{T/T}$= 1 - $\Sigma\pi_{a/b}$). Regarding the error model, the definitions of ADO, ERR and P (*N* | *M*) are the same as for the GT16, except for P (*a*|*b* | *aa*) = (1 – δ) * ⅓ ε.

In simulations, this (wrong) reversibility assumption did not affect performance; there was no decrease in accuracy, and the calculations were ~2X faster than for the GT16 model (see Fig. S22).

Supplementary Note 3. Standard phylogenetic likelihood calculations on DNA sequence alignments

Let us consider how we compute the phylogenetic likelihood on a standard multiple DNA sequence alignment (MSA). For further details, we refer the reader to Felsenstein [51,52] and Yang [73]. The calculation of the phylogenetic likelihood for our genotype model (see Section "phylogenetic likelihood" in the main text) is precisely analogous. Given an MSA comprising the sequences under study, a 4x4 instantaneous rate matrix Q, the ability to compute the corresponding 4x4 transition probability matrix P_t, and the stationary frequency vector π for the four nucleotides, we calculate the likelihood as follows.

We assume that MSA sites evolve independently of each other. Hence, given the MSA, the overall likelihood of the tree is the product over the per-site likelihoods. Thus, it suffices to consider how to compute the likelihood for a single MSA site. Given a fixed tree topology, with fixed branch lengths and known inner/ancestral states for one MSA site, we simply compute the per-site likelihood as the product over all transition probabilities along the tree branches, times the stationary frequency of the root state. While the ancestral nucleotide states are typically not known, we can still calculate the likelihood of the site as the sum over the per-site likelihoods for all possible assignments of nucleotides (i.e., sum over all possible evolutionary histories that could have generated the data, given the tree) to the ancestral states of the given tree topology.

While this, at first glance, appears to be computationally intense (e.g., for a tree with two ancestral nodes, there already exist 4^2 distinct possible assignments of nucleotides to inner nodes of the tree), the likelihood on such a tree can be efficiently computed via the so-called Felsenstein pruning algorithm. The key idea of this algorithm is to calculate the likelihood bottom-up, that is, from the tips toward the root of the tree, and to store intermediate results, so-called conditional likelihood vectors (CLVs), at each inner node of the tree (Fig. S16). CLVs essentially summarize the signal stemming from the subtree they root. That is, they tell us how likely it is to observe an A, C, G, or T, given (conditional on) the subtree they represent. Thus, every inner state in our calculations for one single MSA site consists of a vector containing four conditional likelihoods for, A, C, G, and T, respectively. At the tips, we initialize this vector to (1.0, 0.0, 0.0, 0.0) if we have an A, to (0.0, 1.0, 0.0, 0.0) if we have a C, etc. as the nucleotide state is known and assuming that we are not uncertain about its state.

However, under a single, uniform sequencing error ε, for instance, we can initialize the CLV for A at the tip of the tree as (1.0 - 3ε, ε, ε, ε). This flexibility is used for modeling genotype errors as presented in Section 'single-cell genotype errors' of the main text. The Felsenstein pruning algorithm allows us to compute the likelihood of a given tree, with given branch lengths, and given evolutionary rates specified in Q. Now, to obtain the maximum likelihood (ML) score for such a fixed tree topology, we need to optimize the free parameters of the model, that is, the branch lengths and the rates in Q concerning the likelihood using appropriate numerical optimization routines.

Finally, we also need to find the tree topology with the best ML score, which constitutes an NP-hard optimization problem (i.e., there are no algorithms for finding the ML tree in polynomial runtime as a function of the number of sequences involved) [100]. In layman's terms, this means that we are simply not able to find the globally best ML tree, as there are too many possible tree topologies that would have to be scored. For example, for 50 sequences, there already exist 2.84×10^{74} distinct alternative tree topologies. Therefore, to find the tree with the best ML score, we use *ad hoc* heuristic search strategies that search through this enormous tree space in a "clever" way and return a tree with a "good" score. Most ML tree search strategies imply constructing an initial tree and then apply successive changes ("moves") to the tree topology to find a tree with a better ML score. The three fundamental types of tree moves are Nearest Neighbour Interchange (NNI), Subtree Pruning and Re-grafting (SPR), and Tree Bisection and Reconnection (TBR). Indeed, these heuristics do not offer a guarantee that we find the ML tree, although we typically refer to the best tree found as the ML tree. We refer the reader to Stamatakis et al. [101] for further details.

Furthermore, suppose our matrix Q is time-reversible. In that case, we can root our tree at any branch or node and will always obtain the identical analytical likelihood score for all possible rooting locations. This is computationally convenient, as, for a given tree, we do not need to determine the optimal root placement to compute its ML score. Hence, the output of phylogenetic inferences under

time-reversible models is always an unrooted binary tree, as any rooting will be mathematically meaningless.

Adapting the nucleotide substitution model to a model with more states is, in essence, straight-forward, as precisely the same computational procedure can be used to analyze protein data with 20 states, or our model with ten states here (see corresponding sections on the genotype error and likelihood model in the main text).
