# Supplementary material for: CellPhy: accurate and fast probabilistic inference of single-cell phylogenies from scDNA-seq data
Source: Genome Biol. 2022 Jan 26;23:37. doi: 10.1186/s13059-021-02583-w (PMC8790911; doi:10.1186/s13059-021-02583-w)
Supplement: Supplementary file 2 — Additional file 2. Review history. [file 13059_2021_2583_MOESM2_ESM.docx]

Review History

**First round of review**

**Reviewer 1**

**Were you able to assess all statistics in the manuscript, including the appropriateness of statistical tests used?**

Yes

**Were you able to directly test the methods?**

No

**Comments to author:**

The paper presents the development of a cancer-specific model for single-cell DNA sequencing in the popular RaxML phylogenetic framework. In RaxML an MLE-framework is then available to learn the best tree that explains data, and confidence is assessed by the bootstrap (in the Felsenstein style).

I have been waiting for some time now to see the usage of standard substitution-based models in the context of cancer and single-cell sequencing, also to better enforce cancer-specific methods developed in the broad context of cancer phylogenetic inference.

The method of CellPhy is based on a finite-state Markov genotype substitution model that draws on the original work by Tavare (GTR 1986). The method is presented through simulation and assessed on some small example colorectal cancer single-cell DNA sequencing data. The overall conceptual advantage of this new approach seems only apparent, and I think that the method might be improved to perform even more useful computations (e.g., clustering, plus phylogenetic). In general, data adopted in real samples and tests should be better characterised and shown in the paper. The capability of obtaining novel and relevant biological conclusions with this new method is a strong point made by the authors (also in the cover letter), but still unproved in this paper.

Major:

- Aneuploidy: a strong case is missing to explain why aneuploidy cannot be considered with this model, precluding the usability of the approach for cancers with large levels of aneuploidy like lung, ovarian, breast etc. A detailed motivation should be given why the GTR model cannot be extended to consider allele-specific copy numbers, or at least it should be clarified if this is a limit of scDNA data. In that case, integration of CellPhy with allele-specific clonal CNAs from matched bulk sequencing could be adopted; this would help, and I assume that for most-samples with scDNAseq available, CNAs from bulk DNA sequencing would be also available.

- Performance and model: while I am happy to see the functioning of RaxML in the context of single-cell cancer genomics, the improvement proposed by the tool is not astonishing. In test in Figure 1, for instance, CellPhy and SiFit work very similarly. Practically, CellPhy works significantly better in simulations with very high noise (genotype error ~20%, maximum tested). The most convincing test is Figure 3, Simulation 4, but the test description was unclear to me (*). Therefore, it seems fair to say that with current data (mid quality), the best methods across these tests work generally in a similar way. It would be more interesting to enhance the power of substitution-based models with ideas availabe in tree-inference from machine learning. For instance, can you develop a probabilistic clustering model with phylogenetic, around scDNA sequencing data? Allowing the joint inference of proper phylogenies while clustering cells in a rigorous framework would be a major improvement (not just in performance, but as a concept), bringing classical phylogenetic together with clustering. In general, this would also require to think how a "genetic clone" should be formally defined (and therefore measured). The kind of cell split one is interested into is not necessarily just the classification of primary vs metastatic cell, but rather a more complicated intra-clone phylogeny usually inferred via bulk subclonal deconvolution.

(*) what is 30x and 100x, if cells are sequenced at 5x?

- Performance: CellPhy performs an MLE estimation as far as I understand. SCITE - for instance - performs a Bayesian computation (full posterior). Therefore the performance comparison that makes sense is: CellPhy with Bootstrap, against the cost of a Bayesian Monte Carlo sampler. Clearly, the number of bootstrapped trees is a key factor in this respect (100 are as informative as a posterior?). Please clarify how computed the performance (clock time) in your comparisons.

- Data:

- Colorectal data are never shown along the manuscript, which makes it hard to assess if the tree fits are "good", or which one seems "better". Besides bootstrap scores, the reader should be able to inspect the data and get an intuition of "why" cells cluster together, as the authors observe but do not show. This is also related to the luck of a clustering framework in CellPhy, which is a relevant point since the authors often mention that there are "clusters" in the data. As a side note, it is not clear if the data are newly presented in this manuscript, or taken from other papers. The description alludes to the data being generated for this paper, but it is not clear from the authors contribution who performed the experimental part.

- For the CRC case study only 24 cells are sequenced, from a single tumour; these are a small fraction of a tumour mass with usually billion cells. The sample size is so small to raise the question of how much can be representative of the actual heterogeneity of the cancer. In this sense the paper falls short convincing that, with this type of design/ data and associated analysis, new substantial biology can be discovered. With data of a single patient, findings are also limited in scope. I think that it would be important to show how this new type of analysis plus scDNA can help drawing relevant biological conclusions. In the context of CRC, for instance, stem cell rates are mentioned (as a possible explanation of branches length) - can you prove it or at least verify that across multiple patients?

- it is not clear if simulated data - generated with a tool developed by one of the authros - challenges the method. This should be clarified. For simulated data some examples could be shown form Simulations 1-6, possibly clarifying how noise (of all sorts) affect data quality (e.g., hold fix a CellCoal dataset, and apply gradual increasing noise levels, show how the fit changes for your and other algorithms). Some simulated datasets are very small (40 cells?), and a convincing point that the methods are really challenged should be given.

Minor:

- I am not familiar with the GTR model as probably most of the non-phylogenetics community is. More efforts should be given to detail the key mathematical intuition of all the methods and adopted models, especially because the main technical innovation of the paper is the substitution model, but everything stands on standard MLE computations of RaxML and the GTR model. At present, not enough details are provided even in the Supplementary Note 3. That part should be developed particularly; for instance you say that tree-search is NP-hard, then you mention an imbarassely long number (can you read it?) and you conclude by saying that you can root the tree. We know we cannot approach NP-hard problems in polynomial time, but how do you "solve" (approximate?) the NP-hard problem here? If this is a task for RaxML, then at least you could explain intutitively how that is done.

- are there numerical stability issues in the computations involving matrices? is the conditioning of the rate matrix an issue in performing MLE here?

- equation 6, if it's conditioned on the observed it should be X|Y?

- "A branch length represents the mean number of expected mutations per SNV" seems odd.

- The synthetic tests are presented with multiple of boxplot and maybe some more aggregated representation might be adopted.

**Reviewer 2**

**Were you able to assess all statistics in the manuscript, including the appropriateness of statistical tests used?**

Yes

**Were you able to directly test the methods?**

No

**Comments to author:**

In this article, Kozlov et al. introduce CellPhy, a new method for reconstructing cell-lineage trees from single-cell DNA sequencing data. On simulated data, they compare CellPhy to several existing methods demonstrating its advantage in terms of normalized Robinson-Foulds distance. In addition, several real datasets were analyzed and I will provide more comments on it below.

Overall, there are several major issues with this work. I will start with describing the following three:

(i) For no good reason, some results seem non-reproducible. It is hard to validate some claims that were made and a lot of important information is missing.

(ii) poorly described methods, with obvious mistakes in methods description.

(iii) this work largely ignores most of methods in the field of tumor phylogenetics developed in the past 2-3 years.

There are other major concerns that I am having, but I start with the above three since I find them as absolutely not acceptable in any research article of this type submitted for journal or conference review.

I will illustrate (i) and (ii) through concrete examples and will then discuss (iii).

(i) REPRODUCIBILITY CONCERNS

Consider real data analysis section. I will focus on the patient from Leung et al. study published in Genome Research as it has been previously analyzed in several studies. Assume that, for whatever reason, I want to reconstruct these results using CellPhy.

First, it is not made clear which of the two patients from Leung et al. study is analyzed. Instead of simply and clearly stating that it is patient CRC2 (sometimes also identified as CO8 in the original study, but never in the main text), the authors are introducing their own ID for the dataset. I see no good benefit of introducing a new ID for something which already has a well-established ID in the literature. Maybe it can be confused with another ID used here, CRC24, but then just use a different ID for CRC24, or if CRC24 was already used elsewhere then at least tell a reader that CellPhy introduces ID L86 which refers to CRC2 from Leung et al. paper. So, anyone interested in reanalyzing and validating these results is immediately faced with the question of which dataset is analyzed and is forced to go through various sections of the paper and supplementary materials in order to figure this out. Once I realized that the paper does not contain this information at all, I was forced to go to other referenced publications (like SCARLET) and try to find out an answer. Fortunately, SCARLET analyzed only one patient from Leung et al. so I finally resolved the puzzle. Such a very simple thing is made complicated for no reason.

Next, it is claimed that mutations from genome portions not affected by SNVs are considered for Leung et al. metastatic colorectal cancer dataset. Figure 5D in the Leung et al. paper gives copy number profiles of single cells for this patient. Let's take the first mutated gene that CellPhy paper explicitly mentions, which is APC. As can be seen from Figure 5D from Leung et al. the estimated copy number status shows that genomic locus containing APC gene is affected by copy number gain in almost all of the analyzed cells. The set of cells used for mutation calling might be distinct, but it is extremely unlikely that this copy number variation will not be present in them. So, another ambiguity. But ok. Maybe the copy number calls in Leung et al. are wrong and let's speculate that CNVPanelizer used for CNV calling in CellPhy paper might be doing a better job.

Now, let's have a closer look at the data. To ensure reproducibility, I asked the authors to provide their mutation calls and one of the files provided is vcf file L86.CellPhy. I opened this file and Supplementary Table 5.2 of the original publication and searched for mutations that are present in that table (later I also had a look at Supplementary Figure 7 CRC2, but my experience was similar so below I will focus only on Supplementary Table 5.2). There are at least 13 distinct mutations reported in the table. The only ones which I found in the vcf file provided by the authors are some mutations on chromosome 16. So I found that vcf file contains mutations (chr16, 15917267) and (chr16, 31193877). However, it does not contain mutation at (crh16, 27448836) despite the fact that the original study reports this mutation in a very high fraction of single cells, both at primary and metastatic sites. In addition, its variant allele frequency in exome data is very high. Altogether, it seems very unlikely that this is not a true variant. Now, I am puzzled why do we have this inconsistency?

One might argue that the variant is filtered due to copy number variants. However, this variant lies in between variants at positions 15917267 and 31193877. If one checks Figure 5D of Leung et al. it is clear that the entire chromosome 16, except for possibly its end, is affected by copy number variants. So a possible claim that (crh16, 27448836) is filtered due to CNVs, while (chr16, 15917267) and (chr16, 31193877) are not, is not well supported. Regardless of this, the length of chromosome 16 is over 90 million base pairs and all these three variants fall in the first half of it, which is clearly affected by CNVs as per Figure 5D of the original study. Moreover, it is not a single copy gain only reported in Figure 5D, but in about a half of the cells there is a gain of two copies of chromosome-16 region harboring these three loci so there must be a major inaccuracy in copy number calling in the original study if one wants to attribute this to problems in copy number calling.

Next, I checked the correctness of the following claim "... we identified 597 SNVs distributed over the genome portion not affected by CNVs, including most variants detected in the original study ...". I randomly took the following mutations from the provided vcf file:

(chr15, 90350403)

(chr13, 41133620)

(chr8, 38282099)

(chr6, 11321844)

(chr3, 56894382)

and I was not able to find any of them in the Supplementary Materials of the Leung et al. study.

So, trying to make match of mutation calls from the original study with the ones made in CellPhy, I found almost no match on the sample of 13+ mutations. As can be seen from above, on the sample of 5 randomly chosen mutations I had even worse experience finding literally no match when searching in the opposite direction, so I do not know on what basis the above claim is made. Where did the authors obtain Leung et al. mutation calls from? I could not find this explained anywhere in the CellPhy paper and I could not find mutation calls in the original study, except those provided in the .pdf Supplementary Materials file. It was not clear to me whether Supplemental_Scripts.zip contains these variant calls. All in all, the authors must have made a comparison (otherwise the above claim would not be possible), but I cannot find it explained anywhere in CellPhy paper to what they compared. Trying to figure this out myself also failed as illustrated above.

There is also a slight chance that different reference genomes are used between Leung et al. and CellPhy, although such possibility seems unlikely due to the two matches on chromosome 16 mentioned above. In addition, it is clearly stated in the main paper in Leung et al. that reference genome assembly HG19 was used in their work (Methods section, Sequencing Data Alignment and Processing), same as GRCh37 as indicated in CellPhy paper. Nevertheless, even if differences in reference genome coordinates were indeed present between the two, at some point the authors must have made coordinates conversions in order to be able to make the above claim. So in such case the question which naturally arises is "why the same coordinate-system data is not provided in vcf file or why is this not explained?".

In the end, where are copy number calls made by CNVPanelizer for CRC2 (L86) dataset? If they were not made available, what is the reason for omitting to provide such an important information (even after request that I made through Editors a few weeks ago)? I checked L86.zip filed provided and could not find any information on copy number profiling. Is it because of the following answer (can be found in response to the input data request) "AU(iv): For the L86 dataset, there were no SNV calls outside diploid regions, so the filtering step did not alter it." As there might be significant differences between results of CNVPanelizer and the original study in copy number profiling, please provide the inferred copy number profiles regardless of whether SNVs overlap with CNVs or not.

In any case, regenerating the presented results and validating the claims that were made looks at least very difficult, if even possible.

Regardless of the above, the results reported for this patient are not convincing nor they demonstrate any obvious strength and advantage of using CellPhy for interpreting history of cancer evolution for this patient. I find them rather anecdotal, with one of the main arguments being the fact that one other tool (SCARLET) reports similar results. A single metastatic seeding event is indeed more parsimonious but is not a novel insight about this dataset. On the other hand, several previous analyses found some strong support for bridge-mutations that provide evidence for two metastatic seeding events. In other words, as of now, it is not very clear which of the two interpretations is to be trusted more and results presented in this work do not make any significant difference in resolving this open question.

(ii) METHODS SECTION ARE NOT EASY TO FOLLOW AND CONTAIN OBVIOUS MISTAKES

Methods section is written very poorly and, despite my experience and expertise in the field, I found it extremely difficult to follow and at some point I had to give up attempting to understand the whole methodology. I will give only a few examples of the problems that I faced. There are more and this needs very major revision.

Example 1:

Consider the formula (1):

Q = R * diag(pi_X), where X belongs to the 16-element set Gamma.

Since X is an element of Gamma, there are 16 distinct values of X giving in total 16 distinct diagonal matrices pi_X. How can they all, after being multiplied by R, result in Q? I would expect something like

Q_X = R * diag(pi_X), or possibly

Q = R * diag(pi_Gamma)

but the notation, as is used now, left me completely confused.

Example 2:

What is the point of introducing mu=r(G<->T)=1 ? Why is mu always 1?

Example 3:

The authors are even using Python (or some other language) notation in some expressions. For example, at one place they say "b != a" (right after Equation (5)). Do readers have to know particular programming language syntax in order to be able to decrypt the Math lying behind this?

Example 4:

No motivation, no explanation and no reference is given for Equation (3).

Example 5:

Matrix Q_16 is introduced (defined) in Equation (5) but I do not see that it is used anywhere else in the manuscript.

I also do not see formal definitions for some variables and I am left to guess what they mean (e.g., c for number of cells).

The explanation of methods can be made a way simpler and easier to follow if some supporting figures are provided. This is especially relevant for the Section "Model of nucleotide substitution for phased/unphased diploid genotypes". I recommend adding a simple figure with 4-5 SNVs on, say, 2 chromosomes (2*2 = 4 chromosomal copies in total if diploidy is assumed) and clarify what exactly is genotype, which genotypes can be "adjacent" (i.e., obtained one from another by somatic mutations) etc. ? That can make a big difference and make this work more accessible to other method developers. The similar applies to other parts of the Methods section (in particular Equation (12)).

(iii) MOST OF THE RECENT METHOD DEVELOPMENTS ARE IGNORED AND NOT COVERED

The article provides very outdated review of the existing methods for reconstructing tumor phylogenetic trees from single-cell DNA sequencing data. The overall impression that I got after reading the Introduction, especially the second paragraph, is that this was written 2-3 years ago and no significant effort was put to make appropriate updates after that. Anyway, this needs to be fixed. I am aware of over a half dozen methods published since 2018 (e.g., SPhyR) that are completely ignored in this article. Comparison against more relevant tools needs to be made. An example of such tool is ScisTree (doi: 10.1093/bioinformatics/btz676)

To summarize (i), (ii) and (iii), this work first needs very major revision in order to make it reviewable. I urge the authors to carefully read their work before making journal/conference submissions and ensure that reproducing their results is a straightforward task for other scientists, rather than frustrating or even impossible thing to do. It is possible that I am mistaken. I tried to make my criticisms concrete and they can be proven wrong. However, if at least 50% of them are valid, then, in my opinion, this work unfortunately not deserve any further consideration for publication in Genome Biology. This is a serious journal and we must have some standards. For example, how can one have a sentence like this:

"Raw single-cell whole-genome sequencing data from CRC24 have been deposited in the Sequence Read Archive (SRA - https://www.ncbi.nlm.nih.gov/sra) database under the accession code XXXXX." ?

This is not just a simple typo. If the manuscript was read thoroughly just once before the submission, I doubt that this mistake would not be noticed.

Below, I will add several other comments in hope that they will help the authors to make this work better.

In the second paragraph of the Introduction it is stated that SCITE "essentially estimates a mutation tree to which it attaches the cells a posteriori". SCITE does indeed estimate a mutation tree, but I find this sentence somewhat misleading since SCITE can also work directly in the space of rooted leaf-labelled binary trees. Furthermore, this tree-search approach is not solely a theoretical idea mentioned in the publication of SCITE, but it is in fact supported in the available implementation of this tool. For example, see the option -transpose described at https://github.com/cbg-ethz/SCITE.

It is not clear to me why the fact that CellPhy "does not assume any particular genotypic configuration at the root" is worth emphasizing as a strength of the method. What would be a practical application where this "strength" would make some important difference in comparison to, for example, SCITE and root-genotype model used in it?

"keep", "remove" and "missing" genotype calling strategies are introduced abruptly and without a proper explanation (Page 4).

The use of word "strategies" in the sentence "As before, all strategies performed worse with higher levels of ADO ..." (Page 5) is not appropriate since some related methods have multiple distinct strategies for searching tree or distinct strategies for solving optimization problems. Instead of confusing readers and introducing ambiguities, why do not the authors simply use the word "methods" (instead of "strategies"), the same word which is used in the previous sentence?

Page 5, the last paragraph: it is stated that "... under COSMIC trinucleotide mutational signatures 1 and 5 (i.e., assuming an infinite-sites model)."

Taking into account the meaning of "i.e." (abbreviation for the Latin phrase id est, which means "that is"), why does simulating mutations under COSMIC trinucleotide mutational signatures 1 and 5 imply that simulations are carried under the infinite-sites model? Please explain where does this implication come from.

Page 6, the last paragraph: I find the reference from Stegle et al. 2015 for the presence of doublets as very outdated (more time has passed between now and 2015 than between the invent of single-cell DNA sequencing and 2015). In addition, the authors do not mention that there exist computational tools that might help in filtering doublets (maybe this is due to the lack of expertise in the field, so I take it as something minor). An example of such tool is Single Cell Genotyper (see publication "Clonal genotype and population structure inference from single-cell tumor sequencing" published in Nature Methods several years ago). I would also like to bring to the authors attention one recent tool, doubletD (see publication "doubletD: detecting doublets in single-cell DNA sequencing data" that was presented at ISMB 2021, also published in Bioinformatics journal). doubletD appeared this month so missing to cite it is certainly not a part of my criticism. I am also not asking for in depth review of all related methods and papers (in the end, this is not a review article on doublets), but having a single (outdated) reference while ignoring highly related and more relevant and recent publications is also not appropriate. Please note that I appreciate the efforts made to account for doublets noise and I do not ask for additional experiments in which the authors would compare CellPhy's doublet inference accuracy with that of doubletD or similar methods (such experiments can turn out to be very labor intensive while not bringing much to this work as doublets are not its primary focus).

In Discussion section, the first thing that the authors emphasize is that their tool works under the finite sites model in contrast to several alternatives which use models based on infinite sites assumption. First of all, I do not see a strong evidence in the paper that the use of finite sites model brings some major advantages to CellPhy nor I see that it gives insights to the user about which mutations are affected by such events and where do they occur in the tree. Having such information would be of high interest in interpreting reported results. Second, despite the fact that CellPhy can work under finite sites model, filtering of variants from regions affected by copy number changes is performed during real data analysis. Why? Third, it is mostly losses of SNVs (e.g., due to deletions of chromosomal segments that overlap with variant site) that cause violations of infinite sites assumption when working with single-cell data. Due to higher false negative rates of single-cell data, such mutations are in many cases not having a high impact on tree reconstruction because their absence in a portion of tree affected by deletion can be attributed to allelic dropouts without a lot of penalty. Fourth, it is indeed true that Kuipers et al. demonstrated the presence of violations of infinite sites assumption in their Genome Research paper and identifying such mutations together with their placement is useful, but that is not something done in CellPhy. Lastly, several methods publications demonstrated a high degree of robustness when a fraction of mutations are affected by losses, especially when working with several hundreds or thousands of SNVs. So, overall, I do not see a major benefit of CellPhy over the alternatives, which the authors are trying to emphasize. Model-wise perhaps yes, but practical value of this is unclear.

What is a proof that simulations generated in this study are more realistic than simulations in previously published studies (this claim was made in Discussion section)?

I would not recommend adding comparisons to TNT and OncoNEM to the figures in the main text. It is just distracting the attention of readers. It is quite clear that these methods are not suitable competitors and adding their results is not contributing much to this paper. In my opinion it suffices to just briefly mention their limitations in the main text. For example, the running time issues of OncoNEM are well known and were encountered previously. Adding comparisons with ScisTree instead of with these tools will most likely make the whole simulations section much better.

One very important detail, which I already asked in earlier communication, is supporting mutation trees in the output. A good guidance can already be obtained from SCITE, which reports mutation trees in very simple and elegant way. In addition, denoised genotypes of single cells are something that should be reported (i.e., matrix obtained after noise correction with rows representing cells and columns representing mutations, or vice versa). These two can be of tremendous help to cancer scientists. In my opinion, of the three: (i) cell-lineage tree (with leaf labels only); (ii) mutation tree; (iii) denoised genotypes matrix, the cell-lineage tree is the least useful. It can reveal some clustering of cells, but it is typically mutational history that is more relevant and important in studying cancer. I am aware that what the authors report is not always solely a cell-lineage tree, and it does provide a sort of mutation tree (which can be obtained by looking at which mutations appear at internal edges) but having mutation tree (as in SCITE) and denoised genotype matrix (as in PhISCS) reported as a clear and separate part of the output would add a lot and increase chances of this tool being used in practice. One needs to keep in mind that the potential end users of this tool are not necessarily experts in the domain, and they need simple and easy-to-interpret outputs. They should not be required to write conversion scripts in order to obtain the output in one of the most widely used formats (i.e., mutation trees or denoised genotype matrix), but CellPhy should provide outputs in such format. I am also aware of tutorial https://github.com/amkozlov/cellphy/blob/master/doc/CellPhy-Tutorial.pdf and it is clear to me that authors are familiar with some of these and made efforts in this direction (e.g., Section 5 in the tutorial), but having the above two formats in the output would be very valuable. Note that, if violations of infinite sites assumption are supported, then mutation tree format used in SCITE might need some slight extension because mutation id cannot be used as a synonym for node id. One approach is to use something similar to parent vector representation of mutation tree as used in SCITE, where each node could be represented by (node id, label, parent id) (with root node being a small exception). Alternatively, one can have one file, where each line is of the format (node id, label), which encodes labeling of nodes of the tree. The tree structure can be kept in another file where each line is of a format (parent id, child id) and encodes edge of the tree.

Note that, in addition to enabling clear reporting of mutation trees, CellPhy should also be evaluated and compared to other methods based on measures of tree inference accuracy that focus on placement of mutations. Robinson Foulds measure can be very sensitive to outliers so complementing it with other measures would make experimental section stronger and more convincing.

I also asked for simulated data. While it suffices to provide scripts for generating simulated data, I definitely appreciate the efforts made by the authors to provide all files. Please note that my goal is certainly not to go through every single simulation and try to hunt for mistakes. And I do not have time nor computational resources for doing that. In the end, mistakes are inevitable and all of us make them. But, as I have tried to illustrate above (especially in the first part of the review), what I have seen here is very different from just simple mistakes that we all make. I wish the authors good luck with this work and hope that these suggestions will help them improve the paper.

**Authors Response**

**Point-by-point responses to the reviewers’ comments:**

Reviewer #1:

The paper presents the development of a cancer-specific model for single-cell DNA sequencing in the popular RaxML phylogenetic framework. In RaxML an MLE-framework is then available to learn the best tree that explains data, and confidence is assessed by the bootstrap (in the Felsenstein style). I have been waiting for some time now to see the usage of standard substitution-based models in the context of cancer and single-cell sequencing, also to better enforce cancer-specific methods developed in the broad context of cancer phylogenetic inference. The method of CellPhy is based on a finite-state Markov genotype substitution model that draws on the original work by Tavare (GTR 1986). The method is presented through simulation and assessed on some small example colorectal cancer single-cell DNA sequencing data. The overall conceptual advantage of this new approach seems only apparent, and I think that the method might be improved to perform even more useful computations (e.g., clustering, plus phylogenetic). In general, data adopted in real samples and tests should be better characterised and shown in the paper. The capability of obtaining novel and relevant biological conclusions with this new method is a strong point made by the authors (also in the cover letter), but still unproved in this paper.

*We appreciate the comments. Most likely, we did not stress sufficiently that CellPhy’s model is not cancer-specific but somatic-specific, so it can be used to build phylogenies for any kind of somatic cells (in fact, also for clone genotypes, if available). Also, the main aim of CellPhy is the estimation of the cell phylogeny, which can be leveraged to carry out a variety of evolutionary analyses. In this regard, we believe that the CellPhy model is a clear step forward. Cell clustering is substantially more reduced in evolutionary scope, and efficient tools exist for this specific task. We are not aiming for cancer mutation trees but the estimation of the genealogy of any kind of (asexually reproducing) cells. Also, please note that this manuscript belongs to the Method category, and therefore its focus is on the model and the software, not on the data analysis. The empirical analyses are examples of potential applications, but we understand that their interpretation has to be clear and make sense. In the revision, we have lowered the tone of our claims about the novelty and relevance of the biological conclusions. Below we address each comment in turn.*

Major:

Aneuploidy: a strong case is missing to explain why aneuploidy cannot be considered with this model, precluding the usability of the approach for cancers with large levels of aneuploidy like lung, ovarian, breast, etc. A detailed motivation should be given why the GTR model cannot be extended to consider allele-specific copy numbers, or at least it should be clarified if this is a limit of scDNA data. In that case, integration of CellPhy with allele-specific clonal CNAs from matched bulk sequencing could be adopted; this would help, and I assume that for most samples with scDNAseq available, CNAs from bulk DNA sequencing would be also available.

*The inclusion of aneuploidy in our model is beyond the scope of this manuscript as this would imply not only major work but an entirely new project that might require years if appropriately attempted. From a phylogenetic point of view, copy number variation is very different from single-nucleotide variation, most notably because of the lack of state independence in the former, as copy number variants can overlap. In addition, copy number data is typically not phased. While this might not be a fundamental problem when there are few alternatives (as in the diploid case), it would be when the number of potential phases increases. Also, to extend the GTR model, we would need to obtain allele-specific copy numbers for each cell. Furthermore, the number of genotypic states would explode, complicating computations or even yielding them intractable. In addition, we would need to introduce more parameters for gains and losses. Neither of these tasks is trivial. Please note that no phylogenetic Markov models exist for joint SNVs/CNVs single-cell data, for a good reason. Indeed, this might be an unprecedented challenge that we and others might tackle in the future. We have included a statement about this in the Discussion.*

Performance and model: while I am happy to see the functioning of RaxML in the context of single-cell cancer genomics, the improvement proposed by the tool is not astonishing. In test in Figure 1, for instance, CellPhy and SiFit work very similarly. Practically, CellPhy works significantly better insimulations with very high noise (genotype error ~20%, maximum tested). The most convincing test is Figure 3, Simulation 4, but the test description was unclear to me (what is 30x and 100x, if cells are sequenced at 5x?). Therefore, it seems fair to say that with current data (mid quality), the best methods across these tests work generally in a similar way.

*We explored both easy (Figure 1 or 2) and more realistic scenarios (Figure 3). We expect that under simple, ideal conditions, all methods will perform more or less well. Please note that CellPhy is not only *consistently* better across most simulations, but it is also faster than most methods (1-2 orders of magnitude faster than SiFit). In Figure 3, the difference in accuracy is quite noticeable (85%-90% CellPhy-GL16 vs. 65-70% others) even for moderate error levels (ADO 10%, AMP 5%). Here, 5X, 30X, and 100X are the simulated average sequencing depths (plots for 30x and 100x are shown in Figures S7 and S8). For whole-genome scDNA-seq data, 5X per cell is already expensive, so this is the most realistic depth nowadays. Furthermore, CellPhy accuracy improves substantially with an increasing number of SNVs, giving it an obvious advantage over competing methods for large data sets (please see Figure 4). This advantage is not suprising since the maximum likelihood method is statistically consistent, i.e., it converges to the true tree provided sufficient data.*

It would be more interesting to enhance the power of substitution-based models with ideas available in tree-inference from machine learning. For instance, can you develop a probabilistic clustering model with phylogenetic, around scDNA sequencing data? Allowing the joint inference of proper phylogenies while clustering cells in a rigorous framework would be a major improvement (not just in performance, but as a concept), bringing classical phylogenetic together with clustering. In general, this would also require to think how a "genetic clone" should be formally defined (and therefore measured). The kind of cell split one is interested into is not necessarily just the classification of primary vs metastatic cell, but rather a more complicated intra-clone phylogeny usually inferred via bulk subclonal deconvolution.

*With CellPhy, we do not aim to cluster cells. Cell clustering can be very useful for some specific purposes (say mutation order in cancer). Still, several efficient methods already exist for this task (e.g., SCITE, B-SCITE, SCG, PhISCS-BnB, ddClone, RobustClone). Instead, the scope of CellPhy is to reconstruct full cell lineage trees (cell tree topology plus branch lengths) because they encode information regarding temporal diversification, demographic history, migration, or adaptation. In other words, we want to infer cell trees that can be used for phylodynamic somatic inference (see for example https://science.sciencemag.org/content/371/6526/eaah6266). We have added some text to the Introduction and Conclusions to clarify this.*

Performance: CellPhy performs an MLE estimation as far as I understand. SCITE - for instance - performs a Bayesian computation (full posterior). Therefore the performance comparison that makes sense is: CellPhy with Bootstrap, against the cost of a Bayesian Monte Carlo sampler. Clearly, the number of bootstrapped trees is a key factor in this respect (100 are as informative as a posterior?). Please clarify how computed the performance (clock time) in your comparisons.

*In our opinion, proper comparisons are those aimed at the same outcome. SCITE, or SCIPhi, do not output posterior probabilities for cell tree branches but a point estimate (ML/MAP) of the cell phylogeny –the same as CellPhy. Note that SCITE computes a full posterior for mutation trees but not for cell trees. None of the competing methods output support values for branches of the cell tree except for TNT. In Figure 5, we measure the time taken by each method to produce a point tree estimate, so we believe this comparison is legitimate. Nonetheless, we agree that it might be interesting also to show how fast is CellPhy + bootstrap. We now report running times for 100 bootstrap replicates in Figure S11. Also, we now explain more clearly in Methods how we measured clock time.*

- Data:

Colorectal data are never shown along the manuscript, which makes it hard to assess if the tree fits are "good", or which one seems "better". Besides bootstrap scores, the reader should be able to inspect the data and get an intuition of "why" cells cluster together, as the authors observe but do not show. This is also related to the luck of a clustering framework in CellPhy, which is a relevant point since the authors often mention that there are "clusters" in the data. As a side note, it is not clear if the data are newly presented in this manuscript, or taken from other papers. The description alludes to the data being generated for this paper, but it is not clear from the authors contribution who performed the experimental part.

*Please note that the CRC24 data set includes 17,851 SNVs, so it might be challenging to display. To address the reviewer’s comment, we now show a genotype matrix for all 126 exonic SNVs identified (otherwise, it is impossible to see anything) in a new supplementary figure (Figure S12).*

*Regarding 'clustering”, tree nodes in a cell phylogeny naturally (i.e., according to cell history) group cells into clades (i.e., monophyletic groups). Thus, from an evolutionary point of view, phylogenetic trees also provide 'clusters”. However, the scope of phylogenies goes beyond a simple 'clustering” or 'classification”, as they include branch length information that is useful for dating or for inferring substitution and demographic rates, for example. It is well known that clustering, at least when based on overall similarity, does not equate to evolutionary history, which is our focus. In many cases, clustering can be grossly misleading, for example, when distinct phylogenetic lineages evolve at different rates.*

*The CRC24 dataset was produced in the Posada lab de novo, along with other datasets, by lab members and technicians (see Acknowledgements), participants in separate manuscripts in preparation focused on CRC evolution.*

For the CRC case study only 24 cells are sequenced, from a single tumour; these are a small fraction of a tumour mass with usually billion cells. The sample size is so small to raise the question of how much can be representative of the actual heterogeneity of the cancer. In this sense the paper falls short convincing that, with this type of design/ data and associated analysis, new substantial biology can be discovered.

*We understand that at first sight, the sample size might seem small, but this is whole-genome scDNA-seq human data, and a dataset such as this one is costly and difficult to obtain. Very few analogous datasets exist. Please note that this dataset is multiregional, and includes both stem and non-stem cells, so the 24 cells might represent distinct lineages that coalesce back in time at a more or less early tumoral stage. Moreover, lumping 24 cells at 5x is 120x, while WGS bulks in cancer are typically 30X (indeed, each read from a bulk sample probably comes from a different cell). Interestingly, the VAF distribution computed from these 24 cells is similar to that obtained from the bulk samples (see https://drive.google.com/file/d/1OQWZ9G-xlKmwB_m1tgXNZQ-NjvQC8fJ2/view), which suggests that there already is a substantial amount of information contained in these 24 cells.*

*In phylogenetics and population genetics, samples are often, if not always, much smaller than the population from which they are sampled. For example, evolutionary biologists initially studied worldwide human migration using only dozens of genomes, which already allowed them to obtain fundamental insights (say migration Out of Africa) that have not changed that much after sequencing thousands of human genomes. The same can be said about any organism on earth; we always use 'tiny” samples. When thinking of evolutionary history, regional sampling is often substantially more critical than the number of individuals sampled. Single-cell and bulk data both have pros and cons. In single-cell data, each cell represents a lineage by definition. With bulk data, we need to make additional assumptions (i.e., clonal*

*deconvolution)	to	identify	evolutionary	lineages	(see*

*https://www.sciencedirect.com/science/article/pii/S2405803317301218).*

With data of a single patient, findings are also limited in scope. I think that it would be important to show how this new type of analysis plus scDNA can help drawing relevant biological conclusions. In the context of CRC, for instance, stem cell rates are mentioned (as a possible explanation of branches length) - can you prove it or at least verify that across multiple patients?

*In the text, we state that the fact that non-stem cells exhibit, in general, longer branch lengths than stem cells might indicate potential differences in evolutionary rate, but we did not speculate about the mechanisms behind it. It could be a different cell division rate or a different mutation rate; we cannot tell.*

*Now,	we	know	that	stem	cells	are	typically	slow-cycling*

*(https://www.ncbi.nlm.nih.gov/pmc/articles/PMC3882194; https://pubmed.ncbi.nlm.nih.gov/11689955). We have added a few references to substantiate this statement.*

It is not clear if simulated data - generated with a tool developed by one of the authors - challenges the method. This should be clarified. For simulated data some examples could be shown form Simulations 1-6, possibly clarifying how noise (of all sorts) affect data quality (e.g., hold fix a CellCoal dataset, and apply gradual increasing noise levels, show how the fit changes for your and other algorithms). Some simulated datasets are very small (40 cells?), and a convincing point that the methods are really challenged should be given.

*If phylogenetic accuracy is not perfect (i.e., is <1.0), then the method is challenged by definition, and this happens in all simulations as soon as we add single-cell noise. Contrary to most previous benchmarks assessing these competing models/tools, here, the simulation model is distinct from the inference model. Our simulation model uses a populational coalescent framework, under exponential growth (which generates more difficult-to-infer trees, with short internal branches), with rate variation among lineages, and with a variety of mutation models that assume or not infinite sites, with different mutational signatures (i.e., time-irreversible and context-dependent models), with a variable mutation rate across sites, and that can produce doublets. The inference framework, CellPhy, is a standard maximum likelihood phylogenetic framework extended to diploid genotypes that assumes reversibility, context-independency, etc. We believe that ADO rates of 50% and 10% amplification/sequencing error rates are already very high and therefore challenging. Previous benchmarks of cell phylogeny methods are substantially more limited in scope, often with simpler simulation scenarios, always ignoring nucleotide identities and therefore known mutational signatures, with a limited number of distinct cell genealogies, assuming FN/FP rates instead of ADO/amplification errors (so ignoring the latter can produce FN), or with very few replicates.*

*CellPhy does not have a fit function like the referee might have in mind. Phylogenetic likelihoods are not comparable across different data sets, and noise applied several times to the same true genotypes as suggested would result in distinct datasets. This is why the absolute value of the likelihood does not mean much per se. For example, the likelihood of a given tree will always decrease with a growing number of SNVs, regardless of the SNVs being genuine or noise. In our simulations, we are already showing how accuracy changes across increasing noise levels, and we do this across thousands of possible cell genealogies.*

*As mentioned before, 40 cells is currently a large data set when talking about scDNA WGS data given current sequencing prices. Several competing methods already take a long time even to infer trees with only 40 cells. The number of possible unrooted trees for 40 cells is already vast (exactly 1.009847e+57). In phylogenetic simulations, the main point is to establish the relative performance among competing methods under different scenarios. Absolute performance is less meaningful as simulated data will always be a simplification of real data. In fact, with more cells, the accuracy advantage of CellPhy increases (note that SCIPhI’s constant 0.4 accuracy in Figure 4 is artefactual), so augmenting the number of cells would make CellPhy look even better when compared with the other methods. In any case, please note that we did explore a range of sample sizes up to 1,000 cells, and in these large data sets, CellPhy outperforms every other method.*

*Interestingly, there are methods like PhyDOSE to determine the number of cells to sequence to identify*

*the	“true”	clonal	phylogeny	given	bulk	sequencing	data*

*(*[*https://journals.plos.org/ploscompbiol/article?id=10.1371/journal.pcbi.1008240*](https://journals.plos.org/ploscompbiol/article?id=10.1371/journal.pcbi.1008240)*).*

I am not familiar with the GTR model as probably most of the non-phylogenetics community is. More efforts should be given to detail the key mathematical intuition of all the methods and adopted models, especially because the main technical innovation of the paper is the substitution model, but everything stands on standard MLE computations of RaxML and the GTR model. At present, not enough details are provided even in the Supplementary Note 3.

*We appreciate that some standard methods and equations from statistical phylogenetics might not constitute common knowledge outside the field. Hence, we added clarifications and references, including textbooks, to make the phylogenetic methods more accessible to the general reader.*

That part should be developed particularly; for instance you say that tree-search is NP-hard, then you mention an imbarassely long number (can you read it?) and you conclude by saying that you can root the tree. We know we cannot approach NP-hard problems in polynomial time, but how do you "solve" (approximate?) the NP-hard problem here? If this is a task for RaxML, then at least you could explain intutitively how that is done.

*The NP-hard phylogenetic tree search problem is part of the standard computational phylogenetics literature, and its detailed discussion here seems out of scope. In short, we approximate full, NP-hard tree searches via an ad hoc heuristic tree search based on a hill-climbing approach. In the Methods section, we already give a brief, intuitive explanation of the RAxML/CellPhy search algorithm under “Implementation: Phylogenetic tree search” with references for interested readers., e.g., the RAxML-NG paper by Kozlov et al. (2019) (doi:10.1093/bioinformatics/btz305). In any case, we have added some text to Supplementary Note 3 regarding the tree search strategy.*

Are there numerical stability issues in the computations involving matrices?

*So far, we have not detected numerical stability issues despite conducting extensive simulations. Based on our experience with other models in RAxML-NG, such problems are rare.*

Is the conditioning of the rate matrix an issue in performing MLE here?

*Sorry, but we do not understand the meaning of conditioning here. If the reviewer still refers to the numerical stability, say eigenvalues and eigenvectors for matrix exponentiation, these have been generally solved. However, it is true that exceptions occur on special occasions for non-symmetrical rate matrices. Note that CellPhy only uses symmetrical rate matrices.*

equation 6, if it's conditioned on the observed it should be X|Y?

*Left-hand side (likelihood) is conditioned on the observed, but on the right, the conditioning is reversed (Bayes’ theorem). The formula is correct, but we changed text and notation slightly for clarity.*

"A branch length represents the mean number of expected mutations per SNV" seems odd.

*Sorry, we meant that a branch length represents the “number of expected mutations per SNV along the corresponding branch”. We have eliminated this statement and moved the definition of branch length to the previous section (just before equation 3).*

The synthetic tests are presented with multiple boxplots and maybe some more aggregated representation might be adopted.

*We thought about this, but we did not come up with a better way of representing performance. Boxplots are helpful to show the variance, for example.*

Reviewer #2:

In this article, Kozlov et al. introduce CellPhy, a new method for reconstructing cell-lineage trees from single-cell DNA sequencing data. On simulated data, they compare CellPhy to several existing methods demonstrating its advantage in terms of normalized Robinson-Foulds distance. In addition, several real datasets were analyzed and I will provide more comments on it below. Overall, there are several major issues with this work. I will start with describing the following three:

(i)	For no good reason, some results seem non-reproducible. It is hard to validate some claims that were made and a lot of important information is missing.

(ii)	poorly described methods, with obvious mistakes in methods description.

(iii)	this work largely ignores most of methods in the field of tumor phylogenetics developed in the past 2-3 years.

There are other major concerns that I am having, but I start with the above three since I find them as absolutely not acceptable in any research article of this type submitted for journal or conference review. I will illustrate (i) and (ii) through concrete examples and will then discuss (iii).

*We appreciate the comments, but we instead believe that these are mostly presentation issues easily solved or matters of distinct opinion and style. Below we address each comment in turn.*

(i) REPRODUCIBILITY CONCERNS

Consider real data analysis section. I will focus on the patient from Leung et al. study published in Genome Research as it has been previously analyzed in several studies. Assume that, for whatever reason, I want to reconstruct these results using CellPhy.

First, it is not made clear which of the two patients from Leung et al. study is analyzed. Instead of simply and clearly stating that it is patient CRC2 (sometimes also identified as CO8 in the original study, but never in the main text), the authors are introducing their own ID for the dataset. I see no good benefit of introducing a new ID for something which already has a well-established ID in the literature. Maybe it can be confused with another ID used here, CRC24, but then just use a different ID for CRC24, or if CRC24 was already used elsewhere then at least tell a reader that CellPhy introduces ID L86 which refers to CRC2 from Leung et al. paper. So, anyone interested in reanalyzing and validating these results is immediately faced with the question of which dataset is analyzed and is forced to go through various sections of the paper and supplementary materials in order to figure this out. Once I realized that the paper does not contain this information at all, I was forced to go to other referenced publications (like SCARLET) and try to find out an answer. Fortunately, SCARLET analyzed only one patient from Leung et al. so I finally resolved the puzzle. Such a very simple thing is made complicated for no reason.

*We now explicitly identify L86 in the text as coming from patient CRC2 in Leung et al. Note that the L86 dataset analyzed here corresponds to a subset of 86 cells that were initially genotyped with the T1000 platform. While the original CRC2 set comprises 182 cells, 114 correspond to healthy cells. Since our main goal was to explore the evolutionary relationship of the tumor cells in this particular example, and several of the methods/tools compared are slow, we decided only to include a few (n = 18) healthy cells.*

Next, it is claimed that mutations from genome portions not affected by SNVs are considered for Leung et al. metastatic colorectal cancer dataset. Figure 5D in the Leung et al. paper gives copy number profiles of single cells for this patient. Let's take the first mutated gene that CellPhy paper explicitly mentions, which is APC. As can be seen from Figure 5D from Leung et al. the estimated copy number status shows that genomic locus containing APC gene is affected by copy number gain in almost all of the analyzed cells. The set of cells used for mutation calling might be distinct, but it is extremely unlikely that this copy number variation will not be present in them. So, another ambiguity. But ok. Maybe the copy number calls in Leung et al. are wrong and let's speculate that CNVPanelizer used for CNV calling in CellPhy paper might be doing a better job.

*The set of cells used for copy-number profiling in Leung et al. is different from the set of cells analyzed in our study. On this basis, instead of relying on Leung et al.’s CNV calls, we decided to re-estimate them using a method specifically designed for targeted sequencing data (CNVPanelizer) and just for the same 86 cells for which we called the SNVs. We have uploaded the output from CNVPanelizer to https://github.com/amkozlov/cellphy-paper.*

Now, let's have a closer look at the data. To ensure reproducibility, I asked the authors to provide their mutation calls and one of the files provided is vcf file L86.CellPhy. I opened this file and Supplementary Table 5.2 of the original publication and searched for mutations that are present in that table (later I also had a look at Supplementary Figure 7 CRC2, but my experience was similar so below I will focus only on Supplementary Table 5.2). There are at least 13 distinct mutations reported in the table. The only ones which I found in the vcf file provided by the authors are some mutations on chromosome 16. So I found that vcf file contains mutations (chr16, 15917267) and (chr16, 31193877). However, it does not contain mutation at (crh16, 27448836) despite the fact that the original study reports this mutation in a very high fraction of single cells, both at primary and metastatic sites. In addition, its variant allele frequency in exome data is very high. Altogether, it seems very unlikely that this is not a true variant. Now, I am puzzled why do we have this inconsistency? One might argue that the variant is filtered due to copy number variants. However, this variant lies in between variants at positions 15917267 and 31193877. If one checks Figure 5D of Leung et al. it is clear that the entire chromosome 16, except for possibly its end, is affected by copy number variants. So a possible claim that (crh16, 27448836) is filtered due to CNVs, while (chr16, 15917267) and (chr16, 31193877) are not, is not well supported. Regardless of this, the length of chromosome 16 is over 90 million base pairs and all these three variants fall in the first half of it, which is clearly affected by CNVs as per Figure 5D of the original study. Moreover, it is not a single copy gain only reported in Figure 5D, but in about a half of the cells there is a gain of two copies of chromosome-16 region harboring these three loci so there must be a major inaccuracy in copy number calling in the original study if one wants to attribute this to problems in copy number calling.

*It is not strange that the set of calls is different because we used a different pipeline to analyze the raw data. Leung et al. relied on GATK for variant calling followed by filtering criteria based on read depth. However, since (i) GATK has not been explicitly developed for single-cell sequencing data, and (ii) several single-cell variant callers have been put forward in recent years, we decided to retrieve the original data available from SRA and applied the SCcaller algorithm to the targeted data for SNV identification. In addition, we applied a series of stringent filters. Specifically, at the single-cell level, we filtered out positions overlapping CN variable regions, sites showing an SO tag other than “True”, as well as variable positions with an alternative read count < 3 or showing an ML genotype estimate above 120 (Phred-scaled), as such uncertainty in the genotype call was usually associated with sites experiencing an apparent disparity in the proportion of both alleles (i.e., allelic bias). Afterward, we applied a “consensus” filter (to only retain sites present in at least one cell and the bulk tumor sample or in two cells), a biallelic filter (to remove non-biallelic positions), and a missingness filter (to remove sites with missing data in more than 50% of the cells). All this is explained in Methods.*

Next, I checked the correctness of the following claim '... we identified 597 SNVs distributed over the genome portion not affected by CNVs, including most variants detected in the original study ...'. I randomly took the following mutations from the provided vcf file:

(chr15, 90350403)

(chr13, 41133620)

(chr8, 38282099)

(chr6, 11321844)

(chr3, 56894382)

and I was not able to find any of them in the Supplementary Materials of the Leung et al. study. So, trying to make match of mutation calls from the original study with the ones made in CellPhy, I found almost no match on the sample of 13+ mutations. As can be seen from above, on the sample of 5 randomly chosen mutations I had even worse experience finding literally no match when searching in the opposite direction, so I do not know on what basis the above claim is made. Where did the authors obtain Leung et al. mutation calls from? I could not find this explained anywhere in the CellPhy paper and I could not find mutation calls in the original study, except those provided in the .pdf Supplementary Materials file. It was not clear to me whether Supplemental_Scripts.zip contains these variant calls. All in all, the authors must have made a comparison (otherwise the above claim would not be possible), but I cannot find it explained anywhere in CellPhy paper to what they compared. Trying to figure this out myself also failed as illustrated above.

*For the sake of simplicity, let’s focus on the mutations shown for the CRC2 patient in Supplementary Fig. 7 of the original publication – as these were the mutations used to generate the SCITE tree shown in the Leung et al. study. Of the 36 mutations shown in this figure, our VCF only contains 9 (affecting LINGO2, IL7R, MYH11, FUS, PTPRD, LRP1B, TSHZ3). Our VCF includes several other variants affecting the same essential genes mentioned in the original study (APC, TP53, NRAS, CDK4, FHIT, SPEN, F8). However, we inadvertently compared gene IDs instead of genomic positions. The reviewer is correct when mentioning that our mutations do not fall in the exact genomic positions as the ones reported by Leung et al.. On this basis, we have re-written the sentence "... we identified 597 SNVs distributed over the genome portion not affected by CNVs, including most variants detected in the original study ..." so it now reads as “we identified 598 SNVs distributed over the genome portion not affected by CNVs, including several variants detected in the original study (e.g., LINGO2, IL7R, MYH11, FUS, PTPRD, LRP1B, TSHZ3), as well as other mutations affecting important driver genes in CRC (e.g., APC, TP53, NRAS).”*

*In any case, we went back to our raw calls to investigate why there were 27 mutations in Leung et al. that do not appear in our set. Interestingly, while we found no evidence of mutation at 12 of the Leung et al. sites (CIITA_16:11002009, ALK_2:29416591, ATR_3:142269072, NR3C2_4:149357051, SPEN_1:16202934,*

*PRKCB_16:24230186,	HELZ_17:65119071,	PIK3CG_7:106508397,	LAMB4_7:107732769,*

*LINGO2_9:28778389, LINGO2_9:28064983, F8_X:1541573788), other six sites (NRAS_1:115258747, EPHB6_7:142566125, IL21R_16:27448836, CDK4_12:58144548, ATP7B_13:52534322, CHN1_2:175779934) presented a “False” SO tag in our calls, hence suggesting weak evidence of being a true somatic*

*mutation. Moreover, three mutations in Leung et al.	(TP53_17:7577548, SPEN_1:16258997,*

*NR4A3_9:102595561) contained more than one alternative allele, so we also filtered them out.*

*Finally, we did identify mutations at the remaining six sites (APC_5:112164646, APC_5:112175328, TOX_8:59851979, STRN_2:37105068, MN1_22:28193785, FHIT_3:60412480). Still, we did not call them (either due to absence of coverage, insufficient alternative reads, or high allelic bias) in more than 50% of the cell population and were therefore discarded by our filters.*

*We added some text in Results regarding this issue.*

There is also a slight chance that different reference genomes are used between Leung et al. and CellPhy, although such possibility seems unlikely due to the two matches on chromosome 16 mentioned above. In addition, it is clearly stated in the main paper in Leung et al. that reference genome assembly HG19 was used in their work (Methods section, Sequencing Data Alignment and Processing), same as GRCh37 as indicated in CellPhy paper. Nevertheless, even if differences in reference genome coordinates were indeed present between the two, at some point the authors must have made coordinates conversions in order to be able to make the above claim. So in such case the question which naturally arises is "why the same coordinate-system data is not provided in vcf file or why is this not explained?".

*As explained above, the differences between the variant sets are related to using different calling pipelines.*

In the end, where are copy number calls made by CNVPanelizer for CRC2 (L86) dataset? If they were not made available, what is the reason for omitting to provide such an important information (even after request that I made through Editors a few weeks ago)? I checked L86.zip filed provided and could not find any information on copy number profiling. Is it because of the following answer (can be found in response to the input data request) "AU(iv): For the L86 dataset, there were no SNV calls outside diploid regions, so the filtering step did not alter it." As there might be significant differences between results of CNVPanelizer and the original study in copy number profiling, please provide the inferred copy number profiles regardless of whether SNVs overlap with CNVs or not. In any case, regenerating the presented results and validating the claims that were made looks at least very difficult, if even possible.

*We have uploaded the output from CNVPanelizer to https://github.com/amkozlov/cellphy-paper.*

Regardless of the above, the results reported for this patient are not convincing nor they demonstrate any obvious strength and advantage of using CellPhy for interpreting history of cancer evolution for this patient. I find them rather anecdotal, with one of the main arguments being the fact that one other tool (SCARLET) reports similar results. A single metastatic seeding event is indeed more parsimonious but is not a novel insight about this dataset. On the other hand, several previous analyses found some strong support for bridge-mutations that provide evidence for two metastatic seeding events. In other words, as of now, it is not very clear which of the two interpretations is to be trusted more and results presented in this work do not make any significant difference in resolving this open question.

*In our opinion, the fact that more methods independently support single seeding does provide valuable insights and more evidence for one side of the debate, even though it might not resolve it completely. For the reasons explained in the SCARLETT paper (the parallel losses of chromosomes 2, 3p, 4, 7, 9, 16, and 22 implied by the Leung et al. tree seem highly unlikely; and all but one “bridge” mutations show evidence of deletions) we argue that a single metastatic event is a more convincing explanation, and the results provided by CellPhy reinforces that. Interestingly, both SCiPHI, which conducts its own calling, and ScisTree, which relies on read counts, rather than binary genotypes, support a single metastatic seeding event.*

(ii) METHODS SECTION ARE NOT EASY TO FOLLOW AND CONTAIN OBVIOUS MISTAKES

Methods section is written very poorly and, despite my experience and expertise in the field, I found it extremely difficult to follow and at some point I had to give up attempting to understand the whole methodology. I will give only a few examples of the problems that I faced. There are more and this needs very major revision.

*We appreciate that some standard methods and equations from statistical phylogenetics might not constitute common knowledge outside the field. Hence, we added clarifications and references, including textbooks, to make the phylogenetic methods more accessible to the general reader.*

Example 1: Consider the formula (1): 0 = R * diag(pi_X), where X belongs to the 16-element set Gamma. Since X is an element of Gamma, there are 16 distinct values of X giving in total 16 distinct diagonal matrices pi_X. How can they all, after being multiplied by R, result in 0? I would expect something like 0_X = R * diag(pi_X), or possibly 0 = R * diag(pi_Gamma) but the notation, as is used now, left me completely confused.

*A diagonal matrix is always defined by a vector of its diagonal elements, so it is a single matrix with 16 stationary frequencies (also stated in the text above formula 1). However, to avoid any possibility of confusion, we changed the notation to:*

*Q = R * diag(pi_A|A, pi_A|C, ..., pi_T|T)*

Example 2: What is the point of introducing mu=r(G<->T)=1 ? Why is mu always 1?

*All this is standard in Markov nucleotide substitution models, developed in the ’70s and ’80s. The time unit in such models is the time required to acquire one mutation, so in this way, branch lengths are scaled in average expected mutations per site. This is done because we can only estimate relative instantaneous substitution rates in the absence of temporal information in real-time units. Please see e.g., Felsenstein’s textbook (2004), which we reference in the first sentence of Methods.*

Example 3: The authors are even using Python (or some other language) notation in some expressions. For example, at one place they say "b != a" (right after Equation (5)). Do readers have to know particular programming language syntax in order to be able to decrypt the Math lying behind this?

*We fixed this typo.*

Example 4: No motivation, no explanation and no reference is given for Equation (3)*.*

*This is standard in Markov models of nucleotide substitution. We have added some references. Please see textbooks e.g., Felsenstein (2004) or Yang (2014, p.4), cited in the text.*

Example 5: Matrix Q_16 is introduced (defined) in Equation (5) but I do not see that it is used anywhere else in the manuscript.

*We used Q_16 to denote the specific Q matrix for the GT16 model. We now changed it to Q for the sake of simplicity.*

I also do not see formal definitions for some variables and I am left to guess what they mean (e.g., c for number of cells).

*We have made the existing definition of c explicit.*

The explanation of methods can be made a way simpler and easier to follow if some supporting figures are provided. This is especially relevant for the Section "Model of nucleotide substitution for phased/unphased diploid genotypes". I recommend adding a simple figure with 4-5 SNVs on, say, 2 chromosomes (2*2 = 4 chromosomal copies in total if diploidy is assumed) and clarify what exactly is genotype, which genotypes can be "adjacent" (i.e., obtained one from another by somatic mutations) etc. ? That can make a big difference and make this work more accessible to other method developers.

*We are unsure what the referee wants to see here, but we have tried to produce a supporting figure (Figure S1).*

The similar applies to other parts of the Methods section (in particular Equation (12)).

*All these are standard methods in statistical phylogenetics available in textbooks. In particular, Equation (12) is part of Felsenstein's pruning algorithm (Felsenstein 1981, Yang 2014 pp.103-107).*

(iii) MOST OF THE RECENT METHOD DEVELOPMENTS ARE IGNORED AND NOT COVERED

The article provides very outdated review of the existing methods for reconstructing tumor phylogenetic trees from single-cell DNA sequencing data. The overall impression that I got after reading the Introduction, especially the second paragraph, is that this was written 2-3 years ago and no significant effort was put to make appropriate updates after that. Anyway, this needs to be fixed. I am aware of over a half dozen methods published since 2018 (e.g., SPhyR) that are completely ignored in this article. Comparison against more relevant tools needs to be made. An example of such tool is ScisTree (doi: 10.1093/bioinformatics/btz676)

*Initially, we included what we believe are the most popular methods nowadays, representing distinct approaches regarding the ISM assumption, the error model, the optimization criterion, the search algorithm, or the statistical paradigm. Now, we expanded the Introduction to describe more methods than the ones initially tested. Also, we have added SPhyR, SASC, and ScisTree to the comparison.*

*We tried SPhyR last year, but it turned out that it required a license for the IBM Cplex Optimizer library that is only free for personal use. To run it under the academic license in a supercomputing facility, one had to buy it, which is very expensive. We have now managed to run it through some simulations, but the program crashes for most datasets. These seem to be memory corruption issues (i.e., an internal bug). Therefore, we could not run this program for the complete set of simulations.*

*SASC is extremely slow and highly inaccurate, so we only ran it partially in Simulation 1. OncoNEM is also very slow and inaccurate, although in this case, we ran it (before) for many months and could complete the entire Simulation 1.*

To summarize (i), (ii) and (iii), this work first needs very major revision in order to make it reviewable. I urge the authors to carefully read their work before making journal/conference submissions and ensure that reproducing their results is a straightforward task for other scientists, rather than frustrating or even impossible thing to do. It is possible that I am mistaken. I tried to make my criticisms concrete and they can be proven wrong. However, if at least 50% of them are valid, then, in my opinion, this work unfortunately not deserve any further consideration for publication in Genome Biology. This is a serious journal and we must have some standards. For example, how can one have a sentence like this:"Raw single-cell whole-genome sequencing data from CRC24 have been deposited in the Sequence Read Archive (SRA - https://www.ncbi.nlm.nih.gov/sra) database under the accession code XXXXX." ?This is not just a simple typo. If the manuscript was read thoroughly just once before the submission, I doubt that this mistake would not be noticed.

*“Accession code XXXX” is a standard way for researchers to indicate that the original data will be made publicly available. We will provide a final accession code upon acceptance and before publication.*

Below, I will add several other comments in hope that they will help the authors to make this work better. In the second paragraph of the Introduction it is stated that SCITE "essentially estimates a mutation tree to which it attaches the cells a posteriori". SCITE does indeed estimate a mutation tree, but I find this sentence somewhat misleading since SCITE can also work directly in the space of rooted leaf-labelled binary trees. Furthermore, this tree-search approach is not solely a theoretical idea mentioned in the publication of SCITE, but it is in fact supported in the available implementation of this tool. For example, see the option -transpose described at https://github.com/cbg-ethz/SCITE.

*We used the -transpose option to obtain the infSCITE trees. We extended the introduction and the methods section accordingly.*

It is not clear to me why the fact that CellPhy "does not assume any particular genotypic configuration at the root" is worth emphasizing as a strength of the method. What would be a practical application where this "strength" would make some important difference in comparison to, for example, SCITE and root-genotype model used in it?

*Conceptually, it is one assumption less. In practice, it has important implications to understand somatic mosaicism and cell history. Most importantly, it allows the estimation of the ancestral states at the most recent common ancestor (MRCA) of the outgroup and the ingroup, whatever these might be (for example, healthy vs. tumor; primary tumor vs. metastasis; stem vs. differentiated cells; epithelial vs. mesenchymal cells; T vs. B lymphocytes; different cell colonies derived from the same cell line, etc.). Overall, we only “know” the states of the MRCA of healthy vs. tumor cells, and in this case, this implies assuming limited somatic evolution in normal tissues. Indeed, somatic evolution is not just cancer, and in many examples mentioned above, we do not know the exact ancestral states. Not enforcing/imposing character states at the root is also essential to study the polarity of change, and more accurate phylogenetic dating, including the estimation of tumor age.*

"keep", "remove" and "missing" genotype calling strategies are introduced abruptly and without a proper explanation (Page 4).

*We explain these strategies in the Methods section. We added a reference in Results to this section.*

The use of word "strategies" in the sentence "As before, all strategies performed worse with higher levels of ADO ..." (Page 5) is not appropriate since some related methods have multiple distinct strategies for searching tree or distinct strategies for solving optimization problems. Instead of confusing readers and introducing ambiguities, why do not the authors simply use the word "methods" (instead of "strategies"), the same word which is used in the previous sentence?

*We replaced “strategies” with “methods”.*

Page 5, the last paragraph: it is stated that "... under COSMIC trinucleotide mutational signatures 1 and 5 (i.e., assuming an infinite-sites model)." Taking into account the meaning of "i.e." (abbreviation for the Latin phrase id est, which means "that is"), why does simulating mutations under COSMIC trinucleotide mutational signatures 1 and 5 imply that simulations are carried under the infinite-sites model? Please explain where does this implication come from.

*We removed “i.e.”*

Page 6, the last paragraph: I find the reference from Stegle et al. 2015 for the presence of doublets as very outdated (more time has passed between now and 2015 than between the invent of single-cell DNA sequencing and 2015). In addition, the authors do not mention that there exist computational tools that might help in filtering doublets (maybe this is due to the lack of expertise in the field, so I take it as something minor). An example of such tool is Single Cell Genotyper (see publication "Clonal genotype and population structure inference from single-cell tumor sequencing" published in Nature Methods several years ago). I would also like to bring to the authors attention one recent tool, doubletD (see publication "doubletD: detecting doublets in single-cell DNA sequencing data" that was presented at ISMB 2021, also published in Bioinformatics journal). doubletD appeared this month so missing to cite it is certainly not a part of my criticism. I am also not asking for in depth review of all related methods and papers (in the end, this is not a review article on doublets), but having a single (outdated) reference while ignoring highly related and more relevant and recent publications is also not appropriate. Please note that I appreciate the efforts made to account for doublets noise and I do not ask for additional experiments in which the authors would compare CellPhy's doublet inference accuracy with that of doubletD or similar methods (such experiments can turn out to be very labor intensive while not bringing much to this work as doublets are not its primary focus).

*We now cite the Zafar et al. 2018 review (doi:10.1016/j.coisb.2017.11.008) focused on scDNA-seq data. We also added a paragraph with references in the Discussion regarding doublet detection.*

In Discussion section, the first thing that the authors emphasize is that their tool works under the finite sites model in contrast to several alternatives which use models based on infinite sites assumption. First of all, I do not see a strong evidence in the paper that the use of finite sites model brings some major advantages to CellPhy nor I see that it gives insights to the user about which mutations are affected by such events and where do they occur in the tree. Having such information would be of high interest in interpreting reported results.

*The use of a finite sites model is one assumption less, is more biologically realistic, and in practice allows the identification of homoplasies. We already provide a script to map mutations to branches as customarily done in phylogenetics.*

Second, despite the fact that CellPhy can work under finite sites model, filtering of variants from regions affected by copy number changes is performed during real data analysis. Why?

*Copy number variation and SNV homoplasies (reversals, parallel point mutations) are different things. We filter apparently-non-diploid regions because our evolutionary model does not consider copy number changes. A copy number change is not the same as a single nucleotide change. Even if they might *apparently* result in the same genotype with the current sequencing technologies, their evolutionary behavior is quite distinct. SNVs and CNVs obey different molecular mechanisms and have distinct evolutionary implications. Therefore they occur at different rates and are not equally reversible. They should never be modeled with the same parameters. It does not make sense to assume a back mutation when in fact, a deletion took place. Indeed reverting a deletion is substantially less likely than reverting a single nucleotide change. Furthermore, that a model is finite-site does not mean that it can adequately handle any amount of homoplasy. In single-cell WGS/WES data, by removing apparently-non-diploid regions, we reduce noise while still maintaining a large number of sites*

Third, it is mostly losses of SNVs (e.g., due to deletions of chromosomal segments that overlap with variant site) that cause violations of infinite sites assumption when working with single-cell data. Due to higher false negative rates of single-cell data, such mutations are in many cases not having a high impact on tree reconstruction because their absence in a portion of tree affected by deletion can be attributed to allelic dropouts without a lot of penalty.

*This might be right for some cancers (still recurrent changes can occur, see https://www.biorxiv.org/content/10.1101/2021.03.29.437407v1), but it is not necessarily true for healthy cells.*

Fourth, it is indeed true that Kuipers et al. demonstrated the presence of violations of infinite sites assumption in their Genome Research paper and identifying such mutations together with their placement is useful, but that is not something done in CellPhy.

*We will consider adding this capability in the future.*

Lastly, several methods publications demonstrated a high degree of robustness when a fraction of mutations are affected by losses, especially when working with several hundreds or thousands of SNVs. So, overall, I do not see a major benefit of CellPhy over the alternatives, which the authors are trying to emphasize. Model-wise perhaps yes, but practical value of this is unclear.

*We believe that being able to work directly with DNA data, taking into account empirical mutational biases, and allowing for multiple hits at the same site might not make a difference in some cases, but it might in others. Identifying convergent and parallel mutations has clear implications for detecting specific driver events or mutational hotspots. All this comes at no additional cost, as in the simulations, we show that performance does not decrease when the data fit the ISM.*

What is a proof that simulations generated in this study are more realistic than simulations in previously published studies (this claim was made in Discussion section)?

*These simulations, contrary to the previous ones, use ACGT diploid genotypes, the almost 20,000 genealogies explored are sampled from a populational coalescent distribution, under exponential growth, with rate variation among lineages, and with a variety of mutation models that either assume or not infinite sites, with different empirical mutational signatures (i.e., time-irreversible and context-dependent models estimated from real data), producing ADO and amplification errors rather than FP and FN events (indeed amplification errors can produce FNs, this is neglected in previous studies).*

I would not recommend adding comparisons to TNT and OncoNEM to the figures in the main text. It is just distracting the attention of readers. It is quite clear that these methods are not suitable competitors and adding their results is not contributing much to this paper. In my opinion it suffices to just briefly mention their limitations in the main text. For example, the running time issues of OncoNEM are well known and were encountered previously. Adding comparisons with ScisTree instead of with these tools will most likely make the whole simulations section much better.

*We have removed OncoNEM from the main text figures, but we will keep TNT as maximum parsimony since it is a standard in phylogenetics and it has already been used in several papers focused on (healthy) cell development. We have added ScisTree results for Simulations 4-6, as this program works with read counts.*

One very important detail, which I already asked in earlier communication, is supporting mutation trees in the output. A good guidance can already be obtained from SCITE, which reports mutation trees in very simple and elegant way.

*There are already many efficient tools for reconstruction mutation trees (say SCITE), and this task is not the focus of CellPhy. We will consider adding this capability in the future.*

In addition, denoised genotypes of single cells are something that should be reported (i.e., matrix obtained after noise correction with rows representing cells and columns representing mutations, or vice versa).

*CellPhy integrates uncertainty in its likelihood calculations but does not work with denoised matrices. Obtaining accurate denoised genotypes with the current CellPhy model might not be that straightforward. We will consider adding this capability in the future.*

These two can be of tremendous help to cancer scientists.

*We want to stress that Cellphy is a tool for somatic evolution, not necessarily cancer. We agree that the features requested would be excellent additions, but right now, we consider them to be beyond the scope of this (already long) manuscript.*

In my opinion, of the three: (i) cell-lineage tree (with leaf labels only); (ii) mutation tree; (iii) denoised genotypes matrix, the cell-lineage tree is the least useful. It can reveal some clustering of cells, but it is typically mutational history that is more relevant and important in studying cancer. I am aware that what the authors report is not always solely a cell-lineage tree, and it does provide a sort of mutation tree (which can be obtained by looking at which mutations appear at internal edges) but having mutation tree (as in SCITE) and denoised genotype matrix (as in PhISCS) reported as a clear and separate part of the output would add a lot and increase chances of this tool being used in practice. One needs to keep in mind that the potential end users of this tool are not necessarily experts in the domain, and they need simple and easy-to-interpret outputs. They should not be required to write conversion scripts in order to obtain the output in one of the most widely used formats (i.e., mutation trees or denoised genotype matrix), but CellPhy should provide outputs in such format.

*We consider the cell lineage tree instead to be the most valuable output of CellPhy. As mentioned before, and in the Conclusions, this representation forms the basis for multiple evolutionary analyses (see https://science.sciencemag.org/content/371/6526/eaah6266).*

I	am	also	aware	of	tutorial https://github.com/amkozlov/cellphy/blob/master/doc/CellPhy-Tutorial.pdf and it is clear to me that authors are familiar with some of these and made efforts in this direction (e.g., Section 5 in the tutorial), but having the above two formats in the output would be very valuable. Note that, if violations of infinite sites assumption are supported, then mutation tree format used in SCITE might need some slight extension because mutation id cannot be used as a synonym for node id. One approach is to use something similar to parent vector representation of mutation tree as used in SCITE, where each node could be represented by (node id, label, parent id) (with root node being a small exception). Alternatively, one can have one file, where each line is of the format (node id, label), which encodes labeling of nodes of the tree. The tree structure can be kept in another file where each line is of a format (parent id, child id) and encodes edge of the tree.

*As explained above, we will consider this request for future versions of the software.*

Note that, in addition to enabling clear reporting of mutation trees, CellPhy should also be evaluated and compared to other methods based on measures of tree inference accuracy that focus on placement of mutations. Robinson Foulds measure can be very sensitive to outliers so complementing it with other measures would make experimental section stronger and more convincing.

*All tree distance metrics have pros and cons. RF is sensitive to outliers; still, this is the standard in phylogenetic benchmarking, and it is the most popular metric. We apply the same metric to all methods, and we cannot envision how this can bias the results towards one method. What matters in these comparisons is the relative performance. As explained above, our focus is not on mutation trees or mutation order but cell history. The manuscript is already quite long, so adding more topics, metrics, results, and figures would be, in our opinion, more confusing than helpful.*

I also asked for simulated data. While it suffices to provide scripts for generating simulated data, I definitely appreciate the efforts made by the authors to provide all files. Please note that my goal is certainly not to go through every single simulation and try to hunt for mistakes. And I do not have time nor computational resources for doing that. In the end, mistakes are inevitable and all of us make them. But, as I have tried to illustrate above (especially in the first part of the review), what I have seen here is very different from just simple mistakes that we all make. I wish the authors good luck with this work and hope that these suggestions will help them improve the paper.

*We thank the referee again for the time taken to review our manuscript.*

**Second round of review**

**Reviewer 1**

The authors have provided answers to my questions. I have some extra minor comments that might help the authors improve this paper.

- it is now more clear that the authors intend to apply CellPhy to all asexually-expanding population of cells, i.e., beyond cancer. It would nice if the authors could at least point and reference studies (beyond cancer) that might benefit from the availability of the CellPhy method.

- Aneuploidy: I understand that including aneuploidy in this framework might be difficult and beyond the scope of this manuscript, but I remain that such a contribution would be game changing, and would stress the authors to think about it for future improvements of CellPhy. Note that there are already at least a couple of allele-specific CNA callers for single-cells, i.e. see from a recent Nature Biotech 2021 paper by the Raphael group, and a biorXiv pre-print from the Shah group.

- Performance and model: the authors claim that the methodological improvements I required (i.e., cell clustering) are more cancer-oriented, and therefore prefer not to include them in their phylogenetics-oriented method. I feel this is a missing opportunity: while I understand that the power of a standard phylogenetic method is to unlock anlaysis that are standard in the classical literature (e.g., coalescence etc.), I would have appreciated an attempt at bridging the world of modern machine learning and phylogenomics closer.

- Performance: I think it was required that the authors reported CellPhy performance with bootstrap. In my opinion they could also show a parallel bootstrap implementation because a multi-core machine is quite a standard type of setup nowadays. In general I am not convinced about author's answer regarding Bayesian computations though. Afaik SCITE outputs a full posterior distribution over trees after MCMC sampling; the choice of showing the MAP tree is to output a single tree. The cells are marginalised against each tree, meaning that all possible assignments of a tree are tested and the average is computed. Since from cell phylogenies we can retrieve a mutation tree ordering, I am presuming that the other direction should be viable too.

- Data: I appreciate new SF12. Can it drawn after inputing missing data and sorted using the classical "Cbio"-alike layout? I would also suggest the authors to include as Supplementary Material and Figures VAFs distributions discussed in AU.1.6. They authors should show the data though, not a KDE of the VAFs, to motivate the quality of the obtained VAFs and how much they are representative of a WG-profile. Alternative profiling methods for bulk-based subclonal deconvolution from VAFs should be used (e.g., I am aware of a new method by the Sottoriva group in Nature Genetics), after calling CNAs from the pseudo-bulk; results from single-sample bulk-based deconvolution could be compared to the clade-based analysis of this paper, to develop further a point regarding evolution in CRCs.

- I appreciate downgrading the statements regarding applications of CellPhy. While a method paper might not need to revolve around novel biological conclusions, a killer application for CellPhy is still missing. I think this is also a missing opportunity, as it might fail convincing experimentalists to generate scDNA data for CellPhy. As opposite, there are 2 recent biorXiv papers by the Sanger that show how single-cell phylogenomics plus coalescence - so something standard - can unravel important biological conclusions regarding clonal hematopoiesis. The authors could be motivated to follow similar example applications.

**Reviewer 2**

I thank the Authors for putting an effort in addressing some of my comments in the revised version of the manuscript.

In my previous review, I questioned the following very explicit claim made by the Authors in the original submission: "Using SC-Caller (Dong et al. 2017), we identified 597 SNVs distributed over the genome portion not affected by CNVs, including most variants detected in the original study (e.g., APC, NRAS, MYH11, LINGO2, IL7R, F8, FUS)."

The Authors admit that this claim was incorrect and mention that they "inadvertently compared gene IDs instead of genomic positions". One of the main problems that I am having with this comes from the fact that in the main manuscript Leung and coauthors state the following: " ... and a second nonsense mutation in the APC tumor suppressor". From here, it is very clear that, according to their mutation calls, some genes were mutated more than once so taking gene ids alone is clearly not sufficient when comparing mutation calls. However, without making the relevant comparisons, the Authors made the claim that seems to be far from being correct. I emphasize that Leung et al. made the above statement in the main manuscript and it is not just a detail "hidden" somewhere in the Supplementary Materials of their publication. Even if the Authors have not read the main manuscript and just checked Figure 6B, which shows the SCITE tree, the use of mutations ids like APC_1, APC_2 in that figure is already an indicator of multiple mutations per gene. I believe that any reasonable further investigation into this would lead to the conclusion that checking the genomic coordinates is required and essential in order to do a proper comparison of mutation calls and make any claims about their similarity.

Next, I do not see any strong evidence for potential novel biological insights that can be gained by the use of CellPhy. For example, on the CRC2 dataset it looks like the most of the difference with the tree published in the original study comes from different mutation calling pipelines that were employed. Furthermore, it is not clear why the tree reported by CellPhy is more plausible and relevant than, for example, the tree reported by ScisTree. Overall the biological application of the tool seems quite limited and parts of the results and the analysis are unreliable.

I find that the Authors unfortunately have not addressed several suggestions that I made and that I believe are important in reaching a broader audience of researchers. As an example of this, it can be of a great importance and value to have a simple genotype matrix with the information about a status (present or absent) of a mutation in a cell. I find reasons for avoiding to provide such output very unclear and ambiguous. The Authors also say "We want to stress that Cellphy is a tool for somatic evolution, not necessarily cancer", although a very large portion of the whole work focuses on applications on cancer, comparisons with tools that are best known for cancer applications etc. This is totally understandable considering the importance of cancer and its somatic evolution. However, after a researcher focused primarily on cancer spends time installing and running the tool, she/he is then faced with several of these challenges in interpreting its output, which can limit the application of the tool. As yet another example, I find the response to my comment about Robinson Foulds metric not very convincing. I reiterate that the measure is very sensitive to outliers and provides very limited support for better performance (on simulated data) compared to the existing methods.

**Authors Response**

**Point-by-point responses to the reviewers’ comments:**

Reviewer #1:

The authors have provided answers (or rebuttals) to my questions. I have some extra minor comments that might help the authors improve this paper.

*Thank you, we really appreciate the comments.*

it is now more clear that the authors intend to apply CellPhy to all asexually-expanding population of cells, i.e., beyond cancer. It would nice if the authors could at least point and reference studies (beyond cancer) that might benefit from the availability of the CellPhy method.

*We have included, in Background and in Conclusions, some references to non-cancer studies:*

*https://www.science.org/doi/10.1126/science.aab1785 https://www.nature.com/articles/s41586-021-03790-y https://www.nature.com/articles/s41586-021-03786-8*

*And please see this paper, already cited in the main text, where different applications of cell phylogenies are discussed: https://www.science.org/doi/10.1126/science.aah6266*

Aneuploidy: I understand that including aneuploidy in this framework might be difficult and beyond the scope of this manuscript, but I remain that such a contribution would be game changing, and would stress the authors to think about it for future improvements of CellPhy. Note that there are already at least a couple of allele-specific CNA callers for single-cells, i.e. see from a recent Nature Biotech 2021 paper by the Raphael group, and a biorXiv pre-print from the Shah group.

*We agree. A statistical model for copy number change would be a fantastic contribution to the field of cancer evolution. However, building a realistic CNA phylogenetic model is an enormous challenge, due to overlapping events, even for allele-specific CNAs (I believe CHISEL still assumes non-overlapping events).*

Performance and model: the authors claim that the methodological improvements I required (i.e., cell clustering) are more cancer-oriented, and therefore prefer not to include them in their phylogenetics-oriented method. I feel this is a missing opportunity: while I understand that the power of a standard phylogenetic method is to unlock analyses that are standard in the classical literature (e.g., coalescence, etc.), I would have appreciated an attempt at bridging the world of modern machine learning and phylogenomics closer.

*Clustering cells implies losing evolutionary information compared to a cell phylogeny, particularly when we consider WES or WGS data, with many mutations. We aim for complete cell genealogy to conduct individual-based phylodynamic and phylogeographic analyses. Machine learning can be very useful for some problems and we would love to hear specific suggestions. So far, it has not led to any substantial advance in the field of phylogenetics.*

Performance: I think it was required that the authors reported CellPhy performance with bootstrap. In my opinion they could also show a parallel bootstrap implementation because a multi-core machine is quite a standard type of setup nowadays.

*CellPhy can indeed take advantage of multicore machines. We initially compared single-core running times, such that the comparison with other programs was as fair as possible. Following the reviewer’s suggestion, we now also show parallel running times for bootstrap analyses in Figure S11.*

In general, I am not convinced about author's answer regarding Bayesian computations though. Afaik SCITE outputs a full posterior distribution over trees after MCMC sampling; the choice of showing the MAP tree is to output a single tree. The cells are marginalised against each tree, meaning that all

possible assignments of a tree are tested and the average is computed. Since from cell phylogenies we can retrieve a mutation tree ordering, I am presuming that the other direction should be viable too.

*We believe SCITE’s MCMC is over mutation trees, not over cell phylogenies. In any case, this third-party methodological detail does not affect our results or conclusions. CellPhy can map mutations to branches of the cell phylogeny –using maximum likelihood ancestral state reconstruction, so this is a probabilistic assignment–, but converting a cell phylogeny into a mutation tree is not unequivocal under a finite-site model. Clearly, the ISM makes things much more manageable.*

Data: I appreciate new SF12. Can it drawn after inputing missing data and sorted using the classical "Cbio"-alike layout?

*We guess the reviewer means “imputing”. CellPhy does not impute missing data. Please note that imputation might be more or less straightforward under an infinite-sites model, but not so under a finite-sites model where the same site can change multiple times.*

I would also suggest the authors to include as Supplementary Material and Figures VAFs distributions discussed in AU.1.6. They authors should show the data though, not a KDE of the VAFs, to motivate the quality of the obtained VAFs and how much they are representative of a WG-profile.

*Done. We now show raw histograms in Figure S13.*

Alternative profiling methods for bulk-based subclonal deconvolution from VAFs should be used (e.g., I am aware of a new method by the Sottoriva group in Nature Genetics), after calling CNAs from the pseudo-bulk; results from single-sample bulk-based deconvolution could be compared to the clade-based analysis of this paper, to develop further a point regarding evolution in CRCs.

*We believe that downstream analyses other than the cell phylogeny and its interpretation are out of scope in this (already long) method paper. In particular, we do not see the point in pooling single-cells to do clonal deconvolution. Why would we want to create a mixture when the individual components are already at hand?*

I appreciate downgrading the statements regarding applications of CellPhy. While a method paper might not need to revolve around novel biological conclusions, a killer application for CellPhy is still missing. I think this is also a missing opportunity, as it might fail convincing experimentalists to generate scDNA data for CellPhy.

*In our opinion, our “killer” application is that CellPhy is more accurate and faster than competing methods and that better cell trees will lead to better downstream biological inferences. We are also experimentalists and have generated quite a bit of scDNA-seq WGS data in our lab.*

As opposite, there are 2 recent biorXiv papers by the Sanger that show how single-cell phylogenomics plus coalescence - so something standard - can unravel important biological conclusions regarding clonal hematopoiesis. The authors could be motivated to follow similar example applications.

*These papers are not “single-cell” but “few-cell-sequencing” (or “single-cell-derived-colony” sequencing (as the Sanger data we do analyze in this manuscript; the Lee-Six et al.). These data types are, conveniently, less noisy as there is no whole-genome amplification. Still, this approach does not work under every scenario. It requires limited somatic variation (so it is applied only to normal tissues), a certain amount of tissue availability (e.g., autopsy, surgery), severe involvement of pathologists, and techniques like laser microdissection. Please note that these papers do not present a phylogenetic method but an application of an existing methodology to excellent data. They typically use simple phylogenetic approaches, assume a constant mutation rate, etc.*

Reviewer #2:

I thank the Authors for putting an effort in addressing some of my comments in the revised version of the manuscript.

*We do appreciate the comments and the time taken to do this review..*

In my previous review, I questioned the following very explicit claim made by the Authors in the original submission: "Using SC-Caller (Dong et al. 2017), we identified 597 SNVs distributed over the genome portion not affected by CNVs, including most variants detected in the original study (e.g., APC, NRAS, MYH11, LINGO2, IL7R, F8, FUS)." The Authors admit that this claim was incorrect and mention that they "inadvertently compared gene IDs instead of genomic positions". One of the main problems that I am having with this comes from the fact that in the main manuscript Leung and coauthors state the following: " ... and a second nonsense mutation in the APC tumor suppressor". From here, it is very clear that, according to their mutation calls, some genes were mutated more than once so taking gene ids alone is clearly not sufficient when comparing mutation calls. However, without making the relevant comparisons, the Authors made the claim that seems to be far from being correct. I emphasize that Leung et al. made the above statement in the main manuscript and it is not just a detail "hidden" somewhere in the Supplementary Materials of their publication. Even if the Authors have not read the main manuscript and just checked Figure 6B, which shows the SCITE tree, the use of mutations ids like APC_1, APC_2 in that figure is already an indicator of multiple mutations per gene. I believe that any reasonable further investigation into this would lead to the conclusion that checking the genomic coordinates is required and essential in order to do a proper comparison of mutation calls and make any claims about their similarity.

*We agree, and that is why we already addressed this comment in the previous revision.*

Next, I do not see any strong evidence for potential novel biological insights that can be gained by the use of CellPhy. For example, on the CRC2 dataset it looks like the most of the difference with the tree published in the original study comes from different mutation calling pipelines that were employed. Furthermore, it is not clear why the tree reported by CellPhy is more plausible and relevant than, for example, the tree reported by ScisTree. Overall the biological application of the tool seems quite limited and parts of the results and the analysis are unreliable.

*Our simulations show that CellPhy is more accurate than the competing methods, sometimes slightly so and sometimes by a large margin –when there is single-cell noise, and when sequencing depth is low.. With the Leung et al data (CRC2) Cellphy infers a different tree than the one published, and this leads to a different biological conclusion, a single metastasis. Because CellPhy is much more accurate than ScisTree (Figs 3-4), its results are more reliable by definition but note that ScisTree also supports a single metastasis scenario. And other papers that consider CNVs also support our conclusions (https://pubmed.ncbi.nlm.nih.gov/32864481/). We already explained the applications of the tool in the text (more accurate, faster, gives support measures, does not need known states at the root, provides branch lengths in units of expected number of substitutions, etc). We cannot discuss whether parts of our results and analyses are unreliable unless the reviewer identifies them and explains why they are unreliable.*

I find that the Authors unfortunately have not addressed several suggestions that I made and that I believe are important in reaching a broader audience of researchers. As an example of this, it can be of a great importance and value to have a simple genotype matrix with the information about a status (present or absent) of a mutation in a cell. I find reasons for avoiding to provide such output very unclear and ambiguous.

*We have addressed most of the numerous points made by this reviewer. As explained before, we do map mutations along the phylogeny, and this is our main interest for downstream phylogenetic applications. Under a finite-site model, genotype imputation is not that straightforward. We believe this paper covers numerous aspects and is already quite long. We will consider the suggestion for the future.*

The Authors also say "We want to stress that Cellphy is a tool for somatic evolution, not necessarily cancer", although a very large portion of the whole work focuses on applications on cancer, comparisons with tools that are best known for cancer applications etc. This is totally understandable considering the importance of cancer and its somatic evolution. However, after a researcher focused primarily on cancer spends time installing and running the tool, she/he is then faced with several of these challenges in interpreting its output, which can limit the application of the tool.

*The applications of a cell phylogeny reach far beyond imputing genotypes. We recommend the following review: https://www.science.org/doi/10.1126/science.aah6266*

As yet another example, I find the response to my comment about Robinson Foulds metric not very convincing. I reiterate that the measure is very sensitive to outliers and provides very limited support for better performance (on simulated data) compared to the existing methods.

*The RF metric has well-known pros and cons, but competing methods are compared on the same basis. The RF article has received 1,465 times citations. Indeed, RF is the most widely used metric in phylogenetics to assess phylogenetic performance. Moreover, the RF metric has been also adapted for mutation trees in cancer: https://almob.biomedcentral.com/articles/10.1186/s13015-021-00188-3.*
